# Transcriptional expression patterns of the cortical morphometric similarity network in progressive supranuclear palsy

**DOI:** 10.1111/cns.14901

**Published:** 2024-08-04

**Authors:** Junyu Qu, Yancai Qu, Rui Zhu, Yongsheng Wu, Guihua Xu, Dawei Wang

**Affiliations:** ^1^ Department of Radiology Qilu Hospital of Shandong University, Qilu Medical Imaging Institute of Shandong University Jinan China; ^2^ Department of Neurosurgery Traditional Chinese Medicine Hospital of Muping District Yantai China; ^3^ Magnetic Field‐free Medicine & Functional Imaging Research Institute of Shandong University Jinan China; ^4^ Magnetic Field‐free Medicine & Functional Imaging (MF) Shandong Key Laboratory Jinan China

**Keywords:** gene expression, imaging transcriptomics, morphometric similarity network, neurotransmitter receptor, progressive supranuclear palsy, transcriptional signatures

## Abstract

**Background:**

It has been demonstrated that progressive supranuclear palsy (PSP) correlates with structural abnormalities in several distinct regions of the brain. However, whether there are changes in the morphological similarity network (MSN) and the relationship between changes in brain structure and gene expression remain largely unknown.

**Methods:**

We used two independent cohorts (discovery dataset: PSP: 51, healthy controls (HC): 82; replication dataset: PSP: 53, HC: 55) for MSN analysis and comparing the longitudinal changes in the MSN of PSP. Then, we applied partial least squares regression to determine the relationships between changes in MSN and spatial transcriptional features and identified specific genes associated with MSN differences in PSP. We further investigated the biological processes enriched in PSP‐associated genes and the cellular characteristics of these genes, and finally, we performed an exploratory analysis of the relationship between MSN changes and neurotransmitter receptors.

**Results:**

We found that the MSN in PSP patients was mainly decreased in the frontal and temporal cortex but increased in the occipital cortical region. This difference is replicable. In longitudinal studies, MSN differences are mainly manifested in the frontal and parietal regions. Furthermore, the expression pattern associated with MSN changes in PSP involves genes implicated in astrocytes and excitatory and inhibitory neurons and is functionally enriched in neuron‐specific biological processes related to synaptic signaling. Finally, we found that the changes in MSN were mainly negatively correlated with the levels of serotonin, norepinephrine, and opioid receptors.

**Conclusions:**

These results have enhanced our understanding of the microscale genetic and cellular mechanisms responsible for large‐scale morphological abnormalities in PSP patients, suggesting potential targets for future therapeutic trials.

## Graphical abstract

MSN in PSP patients was mainly decreased in frontal and temporal cortex but increased in occipital cortex. In longitudinal studies, these are mainly in frontal and parietal regions. The expression pattern associated with MSN changes in PSP involves genes implicated in astrocytes, excitatory, and inhibitory neurons and is functionally enriched in neuron‐specific biological processes. MSN were negatively correlated with the levels of serotonin, norepinephrine, and opioid receptors.

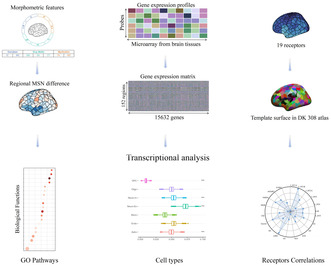

## 1 INTRODUCTION

Progressive supranuclear palsy (PSP) is a rare neurodegenerative disorder with an incidence of approximately 5–7/100,000^1^ and has been confirmed to be associated with the pathological aggregation of tau inclusions.^2^ In addition to the distinct characteristics of supranuclear ophthalmoplegia and balance disorders from falls, additional clinical signs such as cognitive, behavioral, linguistic, and kinetic deficits have been noted.^3^ Although there have been significant attempts, the underlying pathophysiological causes of PSP remain uncertain, owing to the varied impacts of abnormalities and treatments on both brain architecture and functionality.^3^, ^4^, ^5^

Despite their diversity, many new magnetic resonance imaging (MRI) studies have indicated extensive abnormalities in brain structure and function in PSP.^5^, ^6^, ^7^, ^8^, ^9^ To be more precise, previous neuroscientific investigations have shown that reduced functional connectivity was observed between the thalamus and premotor cortex in PSP.^6^ Furthermore, the most severe deficits have been found in hub regions linking the subcortical‐brainstem and prefrontal‐paralimbic modules.^5^ In addition, regarding white matter damage, midbrain atrophy, frontal cortical thinning, and widespread involvement of the main infratentorial and supratentorial tracts have been reported.^4^ Moreover, a recent fronto‐deep gray matter structural connectivity study reported that reductions in nodal degree were found in the hippocampus, frontal pole, pars opercularis, and triangularis of the inferior frontal gyrus at the nodal level.^7^ In addition to the aberrant structural network properties, patients with PSP also exhibit reduced clustering coefficients and weighted degrees in functional networks.^8^ While structural connectomes were computed by using either examining the structural covariance networks in the correlations of morphological measures among subjects^10^, ^11^, ^12^, ^13^ or anatomical connectivity^14^, ^15^ through diffusion‐weighted imaging (DWI) tractography. Nonetheless, the structural covariance method, which relies on a sizeable sample size for precision, overlooks constructing connectomes individually,^16^ and the anatomical connections tend to underestimate the extent of long‐range projections.^17^

Recent advancements in morphometric similarity network (MSN) analysis have significantly enhanced the understanding of macroscale cortical structure.^18^ Instead of assessing the cross‐regional relationship of a singular MRI characteristic among participants, MSNs focus on the cross‐regional association of various morphometric attributes from diverse modalities within an individual. From the perspective of biological relevance, there is a strong correlation between MSN connections and the widespread expression of genes in the brain that are abundant in pathways significant for neurobiology. Cortical regions in MSN with strong connections frequently fall into the same cytoarchitectonic category, a finding corroborated by histological data from nonhuman primates.^19^, ^20^ Furthermore, cortical areas with greater morphometric resemblance tend to have axonal connections among themselves.^21^ In addition, MSN may be perceived as a neuroimaging characteristic that connects alterations in brain structure to transcriptional information.^22^, ^23^ Recent studies have shown that clinical abnormalities of the MSN in diseases such as Parkinson's disease,^24^ schizophrenia,^25^ and major depressive disorder^22^ are strongly correlated with the brain expression of disease‐related genes, revealing the transcriptome and cell pattern vulnerability of the regional brain to neurogenetic diseases.^23^ Although the use of MSN is a reliable and powerful method, morphological differences in PSP have not been investigated.

Recent findings indicate that genes are crucial in the network of the human brain, particularly in forming connections that are both functionally significant and metabolically expensive.^26^, ^27^ Concurrently, genetic elements are crucial in the functioning of brain connectomes,^28^, ^29^ with gene expression atlases across the brain serving as a link between connectomes and transcriptomes.^30^ Access to the comprehensive brain gene expression atlas, derived from the Allen Human Brain Atlas (AHBA) database, has opened up new avenues to explore the link between micro‐level gene expression related to diseases and macro‐level brain changes in diverse neuropsychiatric conditions.^25^, ^31^, ^32^ Researchers have identified transcriptomes associated with human neuroimaging,^33^, ^34^ corroborated by various findings that suggest a link between conserved gene expression and circuits of functional importance.^35^, ^36^, ^37^, ^38^ Combining neuroimaging with gene transcript data has illuminated how microscale structural alterations associated with diseases contribute to widespread brain anomalies in various neuropsychiatric disorders.^23^, ^25^, ^39^, ^40^, ^41^ Consequently, we connect the morphological and functional alterations in the cortex of PSP to gene expression data,^42^, ^43^ aiming to uncover the intricate processes underlying PSP.

**FIGURE 1:**
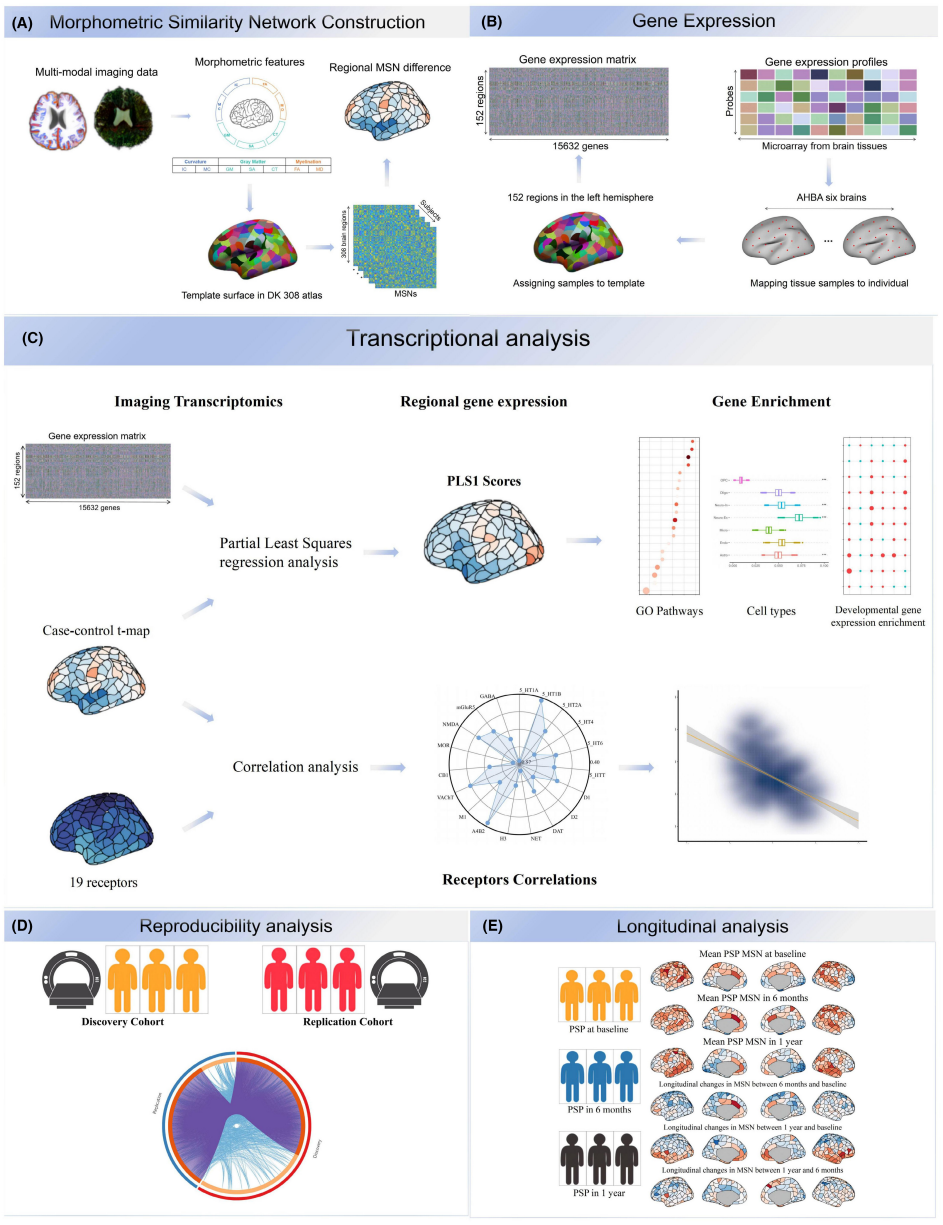
Overview of the study design. (A) Construction of the morphological similarity network. Utilizing the Desikan‐Killiany atlas (D‐K 308), the cortex was partitioned into 308 regions, from which 7 morphological features (GM, SA, CT, IC, MC, FA, MD) were extracted from each T1WI and DTI image, forming the Morphological Similarity Network. (B) Gene expression. By extracting the expression values of each gene in each region (only the left hemisphere) from the AHBA database, we obtain a gene expression matrix. (C) Transcriptomic analysis. PLS regression was performed to link the PSP abnormalities of the MSN with the gene expression data, followed by enrichment analysis of important gene lists from the first component of the PLS (PLS1). Moreover, MSN changes were correlated with 19 types of neurotransmitter receptors. (D) Reproducibility analysis. The use of two independent datasets for reproducibility analysis, confirmed that the changes in MSN are replicable, and that PLS1 captures a large portion of genes and related biological processes that overlap. (E) Longitudinal analysis. The longitudinal cohort of PSP patients was used to compare changes in MSN at baseline, 6 months, and 1 year. AHBA, Allen Human Brain Atlas; CT, cortical thickness; DK, Desikan‐Killiany; FA, fractional anisotropy; GM, gray matter; HC, healthy control; IC, intrinsic (Gaussian) curvature; MC, mean curvature; MD, mean diffusion; MSN, morphometric similarity network; PLS, partial least squares; PSP, progressive supranuclear palsy; SA, surface area.

We hypothesized that there would be notable differences between PSP patients and healthy controls in the MSN and further hypothesized that these differences would be related to gene expression. We combined PSP‐related MSN abnormalities and transcriptional data to elucidate the relationships between molecular mechanisms and structural variations in PSP. First, we describe reproducible PSP‐related MSN abnormalities in two independent cohorts and MSN variations in a longitudinal PSP cohort. Second, we employed partial least squares (PLS) regression to identify specific gene expression patterns spatially associated with PSP regional MSN changes to identify PSP‐related genes. Third, we conducted functional enrichment analysis to infer ontology pathways of PSP‐related genes associated with biological processes. Fourth, we linked aberrant MSN‐related genes to cell types to understand how brain‐wide gene expression and cell types capture molecularly validated anatomical differences in PSP. Finally, we associated PSP‐related MSN changes with PET neurotransmitter receptors to further elucidate our findings. Figure 1 summarizes an overview of the analytical framework.

## 2 MATERIALS AND METHODS

### 2.1 Participants

The discovery cohort comprised 133 individuals, with 51 probable PSPs and 82 healthy controls (HCs). PSP was enlisted from the Four Repeat Tauopathy Neuroimaging Initiative (4RTNI),^44^, ^45^ while HCs entered the study via the Neuroimaging Initiative for Frontotemporal Lobar Degeneration (FTLDNI; http://4rtni‐ftldni.ini.usc.edu/). Eligibility for participation required patients with PSP to satisfy the NINDS‐SPSP criteria^46^ and fall within the age range of 55 to 90 years. The exclusion criteria included (i) being under 18 years of age or above 90 years, (ii) being left‐handed, (iii) having magnetic resonance contraindications, (iv) having natural intracranial lesions, and (v) having substandard image quality evaluated prior to and during the preprocessing of neuroimaging data. Subjects underwent an MRI and a medical assessment including the progressive supranuclear palsy rating scale (PSPRS) rating alongside its components. The longitudinal cohort comes from the same dataset (4RTNI), with some patients lost to follow‐up due to death or inability to leave home. MRI data were collected at 6 months and 1 year after baseline, using the same equipment. After 6 months, 31 patients remained and after 1 year, 16 patients remained. Additional specifics are found in Table S1.

In the replication cohort, participants with PSP (*n* = 53) and HCs who were matched by age and gender (*n* = 55) were selected and examined at Qilu Hospital of Shandong University. For the detailed inclusion and exclusion criteria of this cohort of data, please refer to our previously published article.^9^ Additional information is provided in Table S2. The duplicate studies were approved by the Medical Ethics Committee of Qilu Hospital of Shandong University. Each participant provided their informed endorsement and was aware of the aims, benefits, and potential risks of the study.

### 2.2 Neuroimaging data acquisition and preprocessing

All participants' MRI scan data were gathered via a 3.0‐Tesla MRI scanner. Refer to the Appendix S1 for T1‐weighted imaging data capture. Cortical surface reconstruction involved preprocessing 3D high‐resolution T1‐weighted images using FreeSurfer (v7.3) and performing various techniques, such as skull stripping, tissue segmentation, surface reconstruction, metric reconstruction, and spherical normalization parameter estimation. In addition, the DWI images were preprocessed using FSL (v6.0). See the Appendix S1 for details.

### 2.3 Construction of the morphometric similarity network

The cortical surfaces were segmented into 308 adjoining areas,^18^, ^25^, ^47^ originating from the 68 cortical zones in the D–K atlas.^48^ The segmentation process achieved a roughly uniform scale (~500 mm^2^) in each zone, utilizing a return tracking algorithm^47^ to reduce the impact of differences in parcel dimensions.^18^, ^23^, ^25^, ^49^ The segmented D–K atlas was adjusted to match the surfaces of each participant to achieve a distinct parcellation, which was subsequently interpolated and enlarged to align with their DWI volumes.^18^, ^25^ Seven characteristics from the T1WI and DWI images were collected across every region,^22^, ^25^ encompassing aspects such as surface area, cortical thickness, gray matter volume, Gaussian curvature, mean curvature, fractional anisotropy, and mean diffusivity. Each subject underwent z‐normalization of their morphometric feature vector across various regions to adjust for differences in value distribution among these features.^18^, ^22^, ^25^ Following this, a Pearson's correlation analysis was conducted on the morphometric feature array among each matched cortical area, creating a 308 × 308 MSN for every participant. We compared the MSN values using GLM between PSP patients and the healthy control group, with age, gender, and the interaction of age and gender as covariates.

### 2.4 Case–control analysis of the morphometric similarity network

The regional MSN intergroup variations were analyzed using a General Linear Model (GLM), simultaneously isolating the impacts of age, gender, and their product interaction. Furthermore, for context, regarding changes in the regional MSN within the PSP group, the cortical areas were categorized into two former classifications: the Yeo atlas based on resting‐state networks^50^ and the von Economo atlas, delineated according to cytoarchitectonic standards.^51^ For this purpose, the average MSN values of every area in a specific Yeo network or von Economo class were computed, and a general linear model was used to examine case–control differences in the MSN through regression of these variables. For each region, network, or class, the significance was adjusted using the Benjamini–Hochberg method for false discovery rate (BH‐FDR), ensuring *p* < 0.05.

In the comparison of longitudinal MSN changes of PSP, we compare the differences between baseline, 6 months later and 1 year later. Using the statistical method of paired *t*‐test, due to the small sample size, we use the uncorrected *p* value (*p* < 0.05).

### 2.5 Correlations between the MSN and clinical variables

Average values from brain areas displaying abnormal MSN within the PSP group were gathered to conduct Pearson correlation studies between MSN and clinical factors in PSP patients. The results of the correlation were adjusted using the BH‐FDR method, with a p‐value less than 0.05.

### 2.6 Calculation of regional gene expression

Gene expression information from six brains with 3702 unique postmortem samples was obtained from the AHBA database.^52^ The abagen toolbox (https://www.github.com/netneurolab/abagen) was utilized to handle and align transcriptomic data across 308 segmented brain areas.^53^ In summary, gene expression data preprocessing involves (i) revising annotations between probes; (ii) selecting based on intensity; (iii) choosing probes; (iv) aligning samples with regions; (v) addressing absent data; (vi) normalizing samples; (vii) standardizing genes; (viii) combining samples with regional data; and (ix) pinpointing stable genes. Ultimately, only 15,632 genes were left intact (Supplementary Text). Given that only two of the six brains in the AHBA database contained samples from the right hemisphere, consideration was given exclusively to the left hemisphere. Consequently, an analysis of 152 regions × 15,632 genes utilized a gene expression matrix.

### 2.7 Transcription‐neuroimaging association analysis

A PLS regression analysis^54^ was employed to connect the gene expression profiles of 15,632 genes with the case–control variance in the MSN (*t* values from 152 cortical areas in the left hemisphere). Under the PLS regression framework, the independent variable was the *z*‐score normalized matrix of gene expression (152 regions × 15,632 genes), while the dependent variable was the *z*‐score normalized MSN case–control t vector (152 regions × 1). The PLS elements, a linear amalgamation of weighted gene expression figures, are ordered based on the variance explained between the independent and dependent factors. Consequently, the initial PLS element (PLS1) offers an ideal, simpler depiction of the covariance within complex, high‐dimensional data matrices.^55^ By employing a spatial test for permutation (specifically, the spin test with *n* = 10,000), we assessed whether the explained variability of the PLS component markedly surpassed the random expectations.^56^ Additionally, the bootstrapping technique was used to determine the importance of genes involved in the components (Appendix S1). Only genes with significant results (BH‐FDR *p* < 0.01) were preserved for later examination.

To investigate the associations between PSP‐related gene expression and MSN modifications, we identified 15 genes associated with PSP from the AHBA database^57^ (https://help.brain‐map.org/display/humanbrain/Documentation). To investigate the role of PSP‐related genes in the PLS analysis, we initially extracted coinciding genes from a list of 15 related genes and a background of 15,632 genes. Subsequently, we assessed the correlation between intersecting gene expression and case–control variations in MSN present in the left hemisphere. The threshold for significance was established at *p* < 0.05, adjusted for the false discovery rate (FDR).

### 2.8 Enrichment analyses

Metascape, an autonomous meta‐analysis software (https://metascape.org/) was used to clarify the mechanisms of gene engagement. It is vital to pinpoint uniform possible pathways or networks across various gene lists.^58^ Consequently, a compilation of PLS1− genes from PSP patients was uploaded to the Metascape website to compare the genetic identity and ontology of PLS1− and GWAS genes. The chosen databases included Gene Ontology (GO), Reactome, and the Kyoto Encyclopedia of Genes and Genomes (KEGG). The threshold for determining the importance of the acquired enrichment pathways was 1%, adjusted according to FDR.

### 2.9 Assigning PSP‐related genes to cell types

Postmortem information from five unique single‐cell research papers was collected from cortical samples.^22^ Seidlitz et al.^23^ provided the compilation of specific gene set lists for cells from comprehensive, large‐scale single‐cell analyses of the adult human cortex. Seven different cell types were identified, namely, astrocytes, endothelial cells, microglia, excitatory neurons, inhibitory neurons, oligodendrocytes, and oligodendrocyte precursors (OPCs). The permutation test yielded *p*‐values indicating the count of overlapping genes in every cell type (*p* < 0.05, FDR‐corrected). The objective was to pinpoint the cellular varieties associated with these genes.

### 2.10 Correlations between MSN changes and PET of neurotransmitter receptors and transporters

We utilized the previous work of Hansen et al.^59^ to compute the correlation between PSP‐related MSN changes and 19 PET neurotransmitter receptors (Appendix S1). The *p*‐value is obtained by spin permutation test. The results of the correlation analysis were adjusted by BH‐FDR.

### 2.11 Null model

To mitigate the possible confusing impacts of spatial autocorrelations, a spin test was performed.^56^, ^60^ Creating null Pearson's correlation coefficients is achievable through the random rotation of spherical projections on spatial maps, ensuring that the original spatial relation remains intact. Consequently, in this research, we initiated by rearranging 10,000 spin tests on various cortical areas, resulting in a null distribution, and determined the p_spin_ value by comparing the fraction of null correlation coefficient figures to the actual correlation coefficient values.

### 2.12 Reproducibility analysis

To verify the reliability of our results, we examined the robustness of case–control MSN differences after regressing the influence of total intracranial volume (TIV). A spatial similarity analysis was conducted in the replication cohort, confirming the previously mentioned MSN variations in the discovery cohort. To validate the list of genes associated with PSP obtained through MSN analysis, a meta‐analysis of multiple gene lists was performed (Appendix S1).

## 3 RESULTS

### 3.1 Data samples

For image quality assurance, 9 individuals were excluded because of excessive head movements, 47 with PSP, 80 with HCs in the discovery group, and 51 with PSP, 54 with HCs in the replication group were left for subsequent analysis (Tables S1 and S2). The outcomes we present are derived from the discovery cohort, except when specified differently. No notable differences (*p* > 0.05) were observed between the groups regarding average image quality, age, or gender. To minimize errors in our model, both age and gender were factored into linear models to account for differences between groups.

### 3.2 Case–control differences in the MSN

**FIGURE 2:**
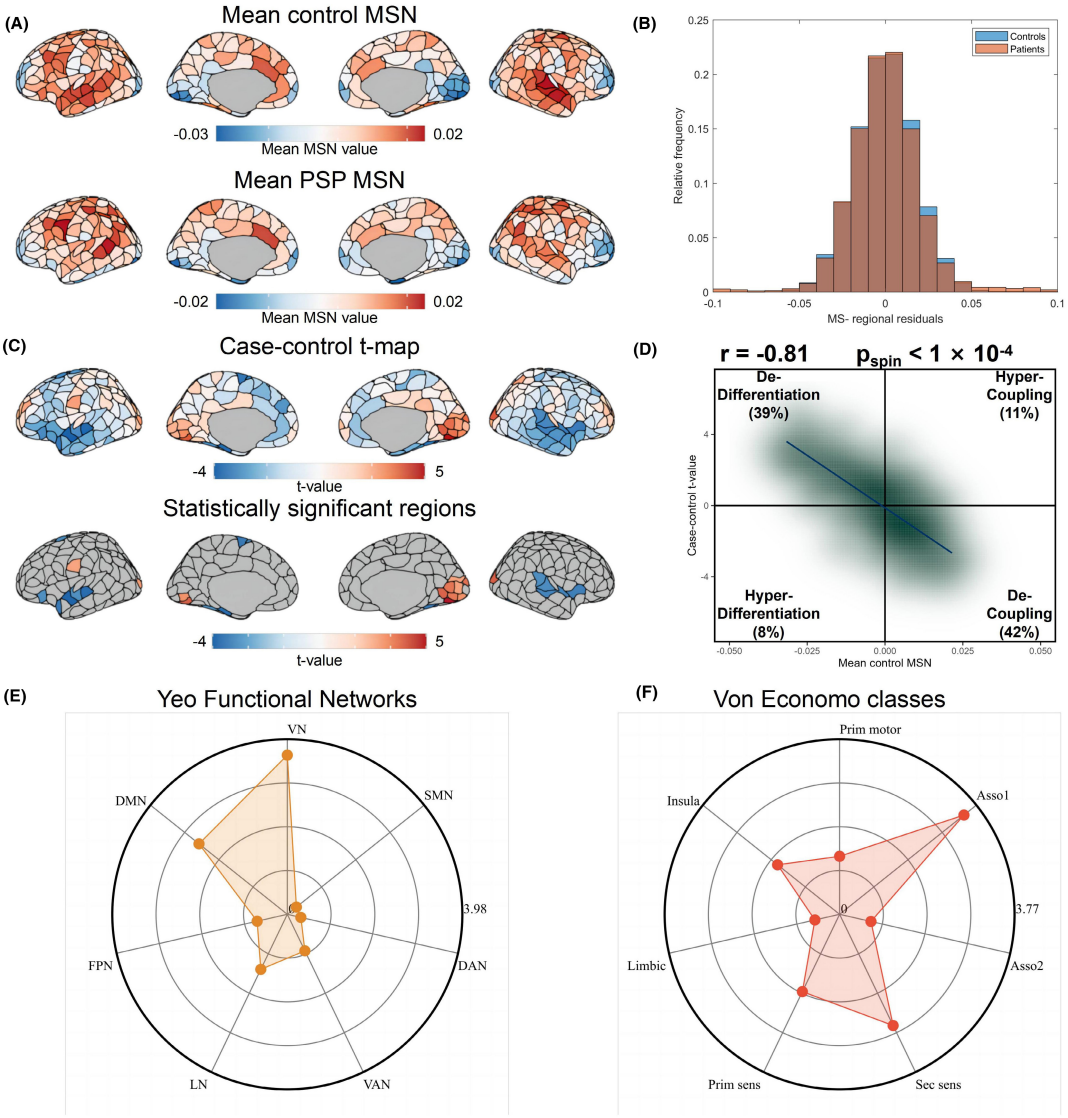
Case–control differences of regional morphometric similarity network. (A) The MSN pattern in patients with PSP and healthy controls. The frontal and temporal lobes exhibited high MSN values, whereas the occipital and somatosensory cortices showed low MSN values. Regions with similar connectivity patterns are shown in similar colors. (B) The histogram shows the distributions of the mean MSN scores in the PSP and HCs groups after regressing out the effects of age, gender, and age × gender. (C) Statistical analyses comparing regions between PSP patients and HCs, with PSP > HCs and PSP < HCs shown in red and blue, respectively (first row, unthreshold; second row, *p* < 0.05, threshold by BH‐FDR correction). (D) A density scatterplot depicting the mean regional MSN scores for healthy subjects (*x*‐axis) and the t‐statistic for the case–control group (*y*‐axis) (*r* = −0.81, *p*_spin_ <1 × 10^−4^). A majority of the cortical areas showed dedifferentiation in 39% and decoupling in 42% of PSP patients. (E, F) The absolute t‐values derived from both the functional community (depicted on the left in Yeo functional networks) and cytoarchitecture (shown on the right in von Economo classes) of the MSN show notable differences, primarily between the visual network and the associate cortex 1 class. Asso1, association cortex1; Asso2, association cortex2; BH‐FDR, Benjamini–Hochberg false discovery rate; DAN, dorsal attention network; DMN, default mode network; FPN, frontoparietal network; HC, healthy control; Insula, insular cortex; Limbic, limbic regions; LN, limbic network; MSN, morphometric similarity network; Prim, motor primary motor cortex; Prim, sens primary sensory cortex; Sec, sens second sensory cortex; SMN, somato‐motor network; VAN, ventral attention network; VN, visual network; PSP, progressive supranuclear palsy.

Figure 2A depicts a cortical representation illustrating the regional morphometric resemblance, which encapsulates the structural distribution of regions displaying both positive and negative similarities in average controls. The findings indicated high positive morphometric similarity in both frontal and temporal cortical regions, contrasted with high negative morphometric similarity when examining the occipital, somatosensory, and motor cortices. This finding validates the reproducibility of the observed regional morphometric resemblance and supports previous studies.^22^, ^25^, ^61^, ^62^ As shown in Figure 2B, after regressing out age, gender, and their interaction, there is a noticeable case–control difference in the average MSN distribution. Furthermore, the t statistics of case–control variations in area‐level MSN across various cortical areas were mapped (Figure 2C). A positive or negative t statistic indicated an increase or decrease in the MSN among PSP patients, respectively, in contrast to the findings in the control groups. Analysis across various regions revealed a reduction in MSN in the left fusiform (part 1), left lateral orbitofrontal (part 4), left parahippocampal (part 1), left superior frontal (part 2 and part 4), left superior temporal (part 5, 6, 7), right fusiform (part 3 and part 5), right superior temporal (part 2 and part5, 6) and right insula (part 4) regions, and an increase in MSN in the left lateral occipital (part 3, part 5 and part7), left lingual (part 4 and part 6), left postcentral (part 4), right cuneus (part 2 and part 3), right lateral occipital (part 1, part 3, part6 and part 7), right lingual (part 2 and part4, 5, 6), right pericalcarine (part 1 and part 3) regions (Table S3). The reduction in the regional MSN in individuals with PSP suggests a diminished morphometric resemblance (or enhanced morphometric distinction) between these regions and other cortical parts, suggesting diminished anatomical links to different, more distinct cortical areas, and the opposite for an increased regional MSN.^22^

There was a notable spatial link between the case–control t‐map and the average regional MSN in the control area (Pearson's *r* = −0.81, *p*_spin_ <0.0001, Figure 2D), suggesting greater differences in case–controls across more interconnected areas.^22^, ^25^ In 42% of the regions, negative regional t‐values and positive mean MSN indicate decoupling in PSP patients compared to HCs, while 39% show positive t‐values, and a negative mean MSN reflects dedifferentiation in PSP individuals in contrast to HCs. This result suggests that areas with extensive connections tend to exhibit greater differences in MSN when comparing cases and controls.

To assess the dependability of our findings, we investigated the impact of TIV on the case–control differences in MSN. Figure S1 shows a strong correlation between the differences in the MSN when TIV was considered in the case–control group and between those without TIV (*r* = 0.9974, *p*_spin_ <1 × 10^−4^).

Additionally, we utilized two earlier categorizations of cortical areas (the Yeo 7 functional networks atlas^50^ and the von Economo cytoarchitecture atlas^51^) to transfer our results to the structural and functional aspects of the brain. According to the Yeo functional networks atlas study, individuals with PSP exhibited a heightened MSN within their visual network (adjusted *p* = 4.33 × 10^−4^, Figure 2E and Table S5). According to the von Economo cytoarchitectural atlas, PSP patients had a reduced MSN in the associate cortex 1 cytoarchitectural category (adjusted *p* = 8.30 × 10^−4^, as shown in Figure 2E and Table S6).

We examined the relationship between PSP‐related alterations in MSN and various clinical symptoms, including PSPRS and its components. No meaningful links were found between MSN measurements of regions that increased or decreased significantly and clinical characteristics (Table S7).

### 3.3 Longitudinal MSN changes in PSP

Utilizing the PSP longitudinal study, we studied the variations of MSN in PSP at three time points—baseline, six months, and one year (uncorrected *p* < 0.05). In longitudinal studies, MSN differences are mainly manifested in the frontal and parietal regions (Appendix S1, Figure S2 and Table S4).

### 3.4 Transcription–neuroimaging associations

**FIGURE 3:**
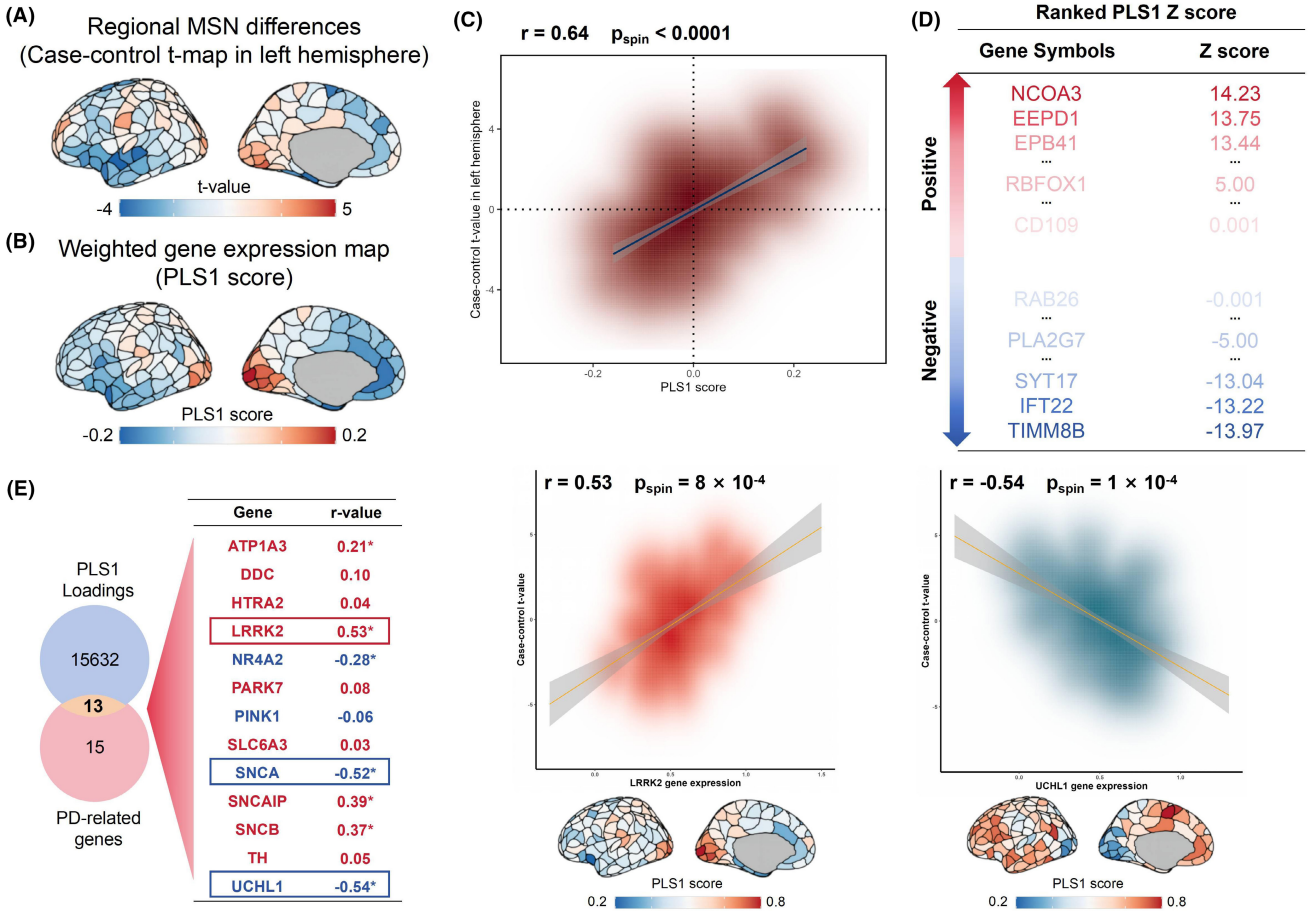
Differences in gene expression profiles related to morphometric similarity network. (A) Case–control t‐map of the regional MSN scores in the left hemisphere. (B) A weighted gene expression map of regional PLS1 scores in the left hemisphere. (C) The density scatterplot illustrates the relationship between regional PLS1 scores and the regional variations in the MSN (*r* = 0.64, *p*_spin_ <0.0001). The gray band represents the 95% confidence interval. (D) Ranked PLS1 genes according to their z score. (E) PD‐related genes from in situ hybridization in the adult human brain correlated with regional changes in MSN. (i.e., LRRK2: *r* = 0.53, *p*_spin_ = 8 × 10^−4^; UCHL1: *r* = −0.54, *p*_spin_ = 1 × 10^−4^; SNCA: *r* = −0.52, *p*_spin_ = 2 × 10^−4^). All *p* values were derived from spin tests and adjusted by FDR. The asterisk represents p values that survived after FDR‐corrected with *p* < 0.05.

The matrix for analyzing brain gene expression was sourced from the AHBA database. In our research, which was limited to two datasets from the right hemisphere, our focus was exclusively on the left hemisphere. Thus, the cerebral gene expression matrix, encompassing 152 regions and 15,632 genes, was utilized in PLS regression to ascertain gene expression patterns linked with the structural arrangement of the MSN for case–control differences (refer to Figure 3A). The primary PLS element (PLS1) accounted for 42.93% of the differences in the MSN case–control discrepancies, surpassing probabilistic expectations (spin test, *p*_spin_ <1 × 10^−4^). The spread of PLS1 scores revealed a gradient in gene expression from front to back (Figure 3B), aligning with findings from an earlier investigation.^22^ There was a positive correlation between the PLS1 gene expression map and the case–control t‐map (*r* = 0.64, *p*_spin_ <1 × 10^−4^, Figure 3C). In line with prior research,^22^, ^25^ our ranking of PLS1's normalized weights was based on the z score assigned to each gene. A total of 3191 genes were recognized as having a substantial impact on PLS1 (*p* < 0.01, BH‐FDR corrected, Figure 3D). Within this group, 1558 genes displayed normalized positive PLS1 weights (PLS1 +), while 1633 genes showed normalized negative PLS1 weights (PLS1−), signifying whether gene expression levels were excessive or insufficient, linked to the MSN fluctuations in either the healthy control group or the PSP group.

In exploring the links between PSP‐linked genetic expressions and MSN regional variations, we initially found 15 genes affected by PSP by examining the term “progressive supranuclear palsy” from the “Gene List” category of the AHBA's in situ hybridization data.^57^ Subsequently, we selected 13 specific genes that coincided with the 15,632 background genes. Among the 15 genes, 13 genes linked to PSP from the AHBA database played a major role in enhancing PLS1 (refer to Table S8) of which nine genes (LRRK2, SNCAIP, SNCB, ATP1A3, DDC, PARK7, TH, HTRA2, and SLC6A3) showed positive correlations with MSN changes, while four genes (UCHL1, SNCA, NR4A2, and PINK1) showed negative correlations (all p_spin_ <0.05, BH‐FDR corrected, Figure 3E and Figure S3).

### 3.5 Enrichment pathways of genes associated with changes in MSN

**FIGURE 4:**
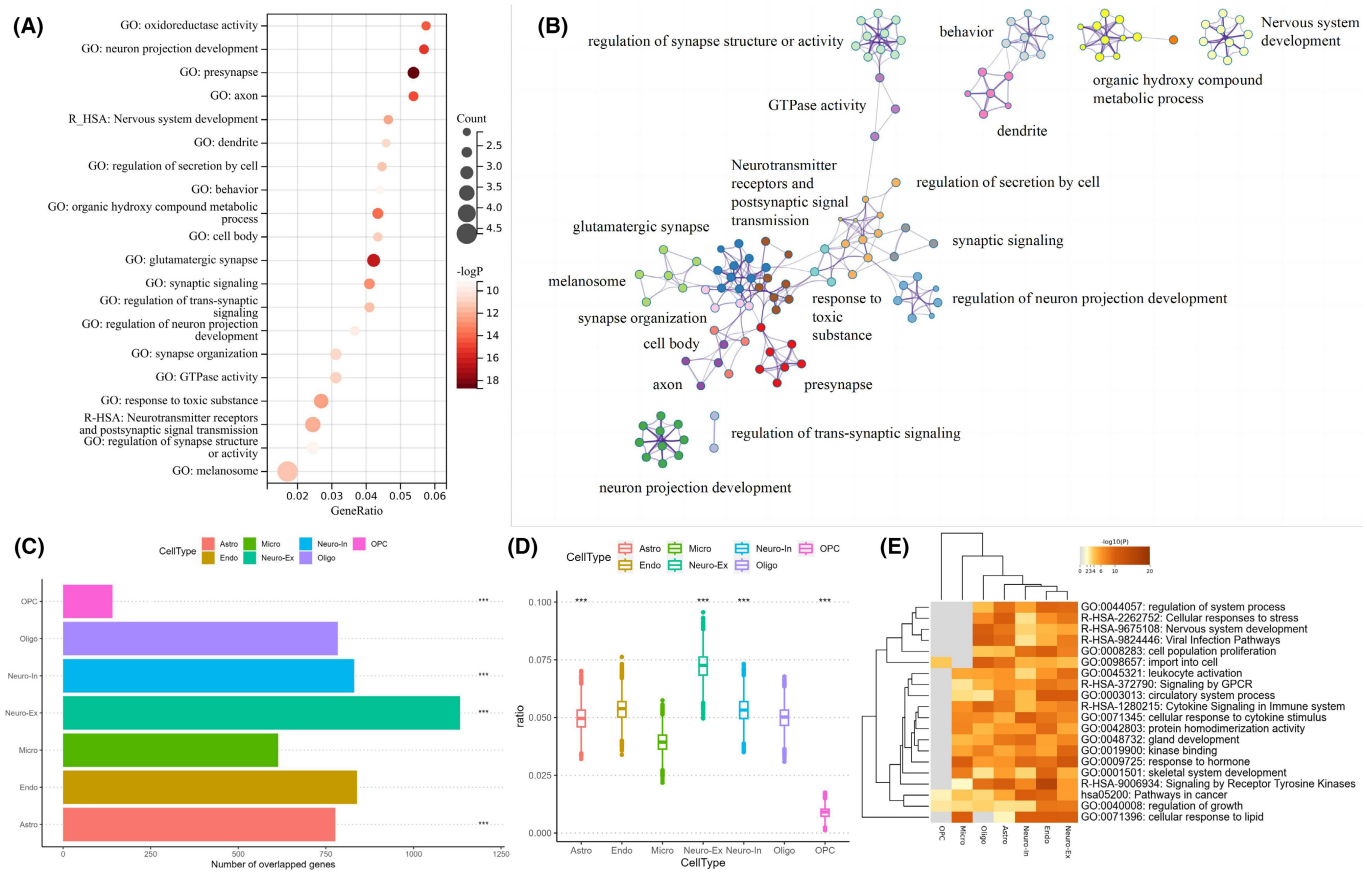
Enrichment analyses of the PLS1 − genes. (A) The bubble plot illustrates the GO and KEGG functional labels for the PLS1 − genes. The bubble size indicates the number of genes overlapping between the PLS1 − gene list and each GO term, reactome gene set, or KEGG pathway. The color bar symbolizes the FDR‐corrected p value. (B) Metascape enrichment network visualization showing the intracluster and intercluster similarities of enriched pathways. Each pathway is shown by a node, with its size corresponding to the number of input genes in the pathway, and different colors indicate responses to different clusters. (C–E) Cell type‐specific changes in the expression of MSN‐related genes. (C) The number of overlapping genes for each cell type (astrocytes: number = 777, *p* < 0.0001; excitatory neurons: number = 1133, *p* < 0.0001; inhibitory neurons: number = 831, *p* < 0.0001; microglia: number = 614, *p* = 0.42; endothelial neurons: Number = 839, *p* = 0.12; oligodendrocyte precursors (OPCs): Number = 141, *p* = 0.17; oligodendrocytes: Number = 784, *p* < 0.0001; all p values were derived from permutation tests adjusted by FDR). An asterisk represents pFDR <0.05. (D) Cell‐type deconvolution was used to identify cell‐type enrichment in the gene sets identified by PLS analysis. The ratio of genes in each gene set preferentially expressed in seven distinct cell types is shown against a null model of a random selection of all genes (boxplots; 10,000 repetitions). *p < 0.05. (E) Gene ontology terms enriched for changes in MSN‐related genes for the cell types. Astro, astrocyte; BH‐FDR, Benjamini–Hochberg false discovery rate; Endo, endothelial; GO, gene ontology; KEGG, Kyoto Encyclopedia of Genes and Genomes; Micro, microglia; Neuro‐ex, excitatory neurons; Neuro‐in, inhibitory neurons; Oligo, oligodendrocyte; OPC, oligodendrocyte precursor; PLS, partial least squares.

We used Metascape for annotations of gene functions and utilized 15,632 genes, each with appropriate brain expression information, as the background. The PLS1 − gene list showed enrichment in various GO biological processes, Reactome gene sets, and KEGG pathways (Figure 4A,B and Figure S4).

To explore identical enrichment pathways linking PLS1− genes to polygenic risk genes for PSP, a meta‐analysis involving multiple genes between PLS1− genes and potential risk genes from PSP genome‐wide association studies (GWAS) was conducted^63^, ^64^, ^65^, ^66^, ^67^ (Figure S5).

### 3.6 Cell–type specific expression associated with changes in MSN

To enhance the accuracy of our research and consider the diversity of brain cells, we divided the PLS1− gene into seven cell types^23^ and conducted gene enrichment studies targeting specific cell types. We found that astrocytes, excitatory neurons, inhibitory neurons, and oligodendrocytes were significantly involved (*p* < 0.0001, FDR correction, Figure 4C–E).

### 3.7 Relationship between MSN in PSP and neurotransmitter receptors

**FIGURE 5:**
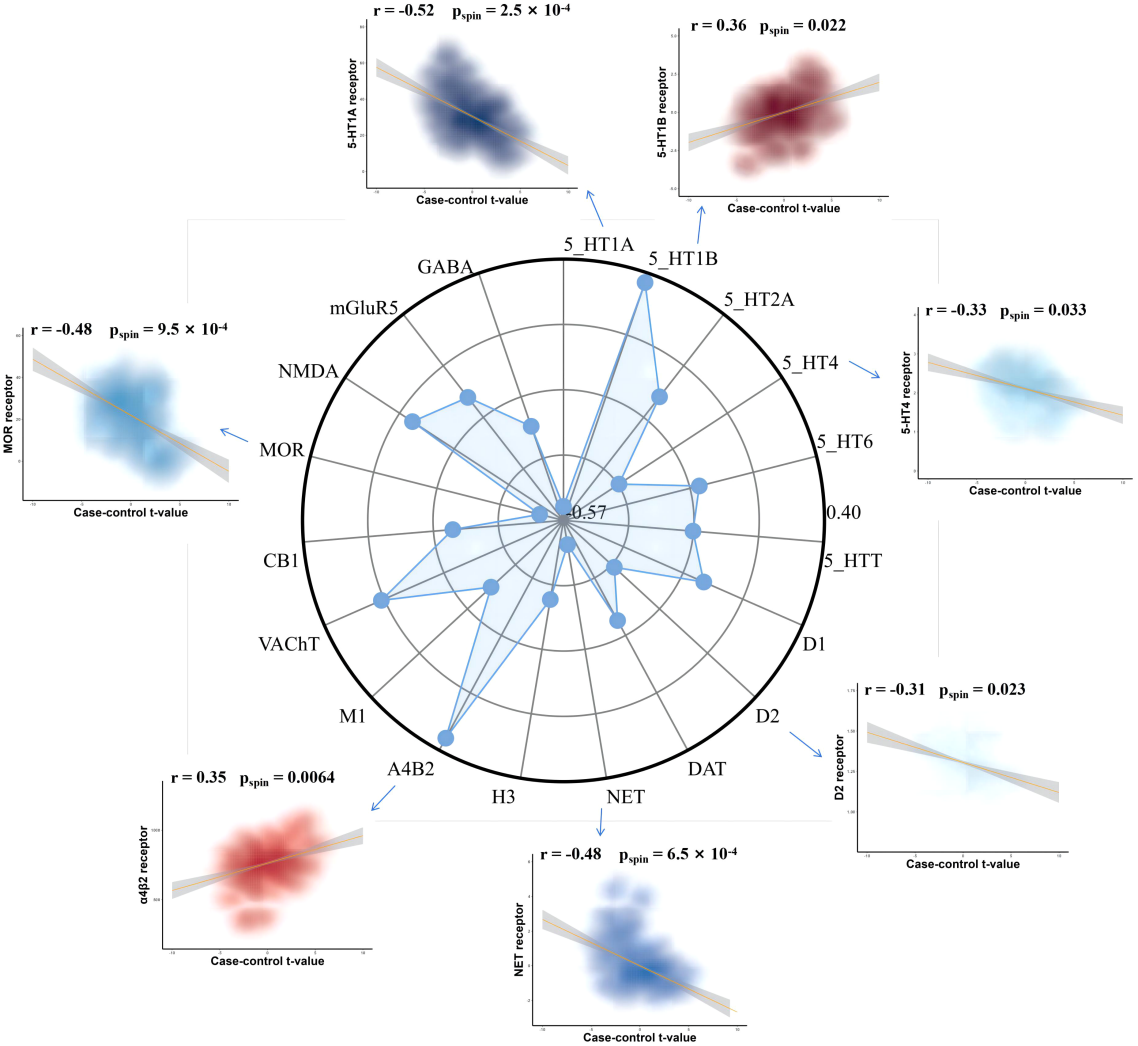
The relationship between PSP‐related changes in MSN and PET neurotransmitter receptors. By mapping the expression profiles of 19 PET neurotransmitter receptors onto the D‐K 308 brain surface and correlating them with differential changes in PSP‐associated MSN cases, we found that 7 neurotransmitter receptors were correlated with MSN changes. Negative correlations included 5‐HT1A, 5‐HT4, NET, MOR, and D2 receptors; positive correlations included 5‐HT1B and α4β2 receptors.

By evaluating the influence of neuromodulatory systems on MSN variance in PSP, our research focused on the relationship between neurotransmitter receptor density patterns and MSN alterations in PSP patients. Our findings indicated the changes of MSN in PSP correlated specifically with a decrease in the D2 receptor, NET receptor, MOR receptor, and two serotonin receptors (5‐HT1A and 5‐HT4), as did increases in the levels of the α4β2 and 5‐HT1B receptors (Figure 5).

### 3.8 Reproducibility analysis of PSP‐related variations in MSN and the transcriptomic profile

We validated the reproducibility of MSN using an independent replication cohort, and the results of the spatial correlation analysis indicated that the MSN pattern is reliable (*r* = 0.52, *p*_spin_ <0.0001; Figure S6).

To validate the gene list related to PSP obtained through MSN analysis, a meta‐analysis of multiple gene lists was conducted, comparing the PLS1− gene lists between the discovery and replication groups. We found a strong overlap between the gene lists of the discovery and replication groups: odds ratio (OR) = 1.46, *p* < 0.0001.

## 4 DISCUSSION

In this study, for the first time, we investigated abnormalities of the MSN in PSP patients and healthy controls. We found a significant association between the cortical pattern of morphometric similarity differences in PSP and the regional transcriptome signatures of PSP‐related genes. First, we found the PLS1− genes, which showed spatial correlations with MSN changes related to PSP, with an enrichment of previously identified PSP‐related genes, and showed ontological terms nearly identical to those found in GWAS studies. Second, these genes play a role in astrocytes and neuronal cells and are functionally enriched in neuron‐specific biological processes related to trans‐synaptic signaling and nervous system development. Third, we investigated the relationship between 19 neurotransmitter receptors and the changes of MSN in PSP and found that 7 neurotransmitter receptor profiles are closely related to MSN variations. Finally, we conducted an exploratory analysis of the longitudinal changes in PSP‐related MSN differences and validated the consistency of MSN changes in replication cohorts, confirming the ontological enrichment of synaptic‐related terms.

Recently, the emergence of complex network theories has significantly enhanced our understanding of how the brain operates and is a broad‐scale neural network, namely, the connectome. In this context, pathology is viewed as the occurrence of irregular links and interactions among various brain areas instead of arising from isolated lesions.^68^ Contrary to standard morphometric networks that build intersubject networks based on a distinct morphological marker of a subject group, the MSN represents morphological similarities across areas from various MRI anatomical markers in each subject. The analysis of morphological resemblances revealed a solid and biologically reliable cortical variation in patients with neuropsychiatric disorders.^69^ The findings revealed a predominant decrease in MSN within frontal and temporal cortical regions, while a significant increase was observed in the occipital cortical zone in PSP patients. Earlier research indicated a greater likelihood of anatomical connections and cytoarchitectural similarities among the morphometrically akin cortical regions.^18^, ^21^ Stemming from this hypothesis, we theorized that the diminished MSN in the frontal and temporal cortices of PSP patients signifies enhanced architectural diversification and diminished interconnectedness of axons between these regions and the broader cortex. Consistent with our results, prior investigations of PSP patients revealed atrophy of the midbrain, a reduction in anterior cortical thickness, and extensive engagement of the primary infratentorial, and supratentorial tracts.^4^ Furthermore, multimodal imaging revealed decreased functional connectivity between the thalamus and premotor cortex in PSP patients^6^ and severe deficits in the hub regions linking the subcortical‐brainstem and prefrontal‐paralimbic modules,^5^ Increased MSN might correlate with adaptive shifts and poor reactions to neurodegenerative mechanisms in PSP patients.

Considering the intricate disease process of PSP, the PSP‐related alterations in MSN seem to be influenced by a range of factors such as genetic, neuronal, molecular, and cellular differences.^63^, ^70^, ^71^ By employing the PLS regression technique to correlate neuroimaging phenotypes with gene expression patterns in the AHBA dataset, we found a group of genes in PLS1 with markedly positive or negative impacts that correlate with the MSN alterations in patients with PSP. Genes included in the PLS1− list showed high activity in the occipital brain regions, with notable enrichment across various pertinent categories. The recognized categories primarily played roles in the development of neurons, axonal direction, and stimulus reactions, suggesting that neuronal deficits and underdevelopment might be potential causes of PSP. Furthermore, the identified UCHL1 gene exhibited the most significant negative correlation and previous research has shown decreased levels of UCHL1 in the cerebrospinal fluid of patients with PSP.^72^ UCHL1 plays a significant role in regulating cellular free ubiquitin levels, oxidative‐reductive status, and the degradation of specific proteins. Loss of UCHL1 function may exacerbate brain damage and neurodegenerative changes.^73^ LRRK2, as the gene exhibiting the strongest positive correlation, is considered to be associated with the survival of PSP patients, and the mechanism of this association may involve the regulation of LRRK2 expression through lncRNAs.^65^ These findings highlight the role of LRRK2 regulation as a potential therapeutic target for disease‐modifying treatments in PSP and related tauopathies, warranting further analysis.

Subsequent research revealed that PLS1− genes play a crucial role in astrocytes, excitatory neurons, and inhibitory neurons, with a notable functional enhancement in neuron‐specific biological functions associated with trans‐synaptic signaling and the development of the nervous system. Astrocytes, the dominant type of glial cell,^74^ are involved in pivotal central nervous system (CNS) development and functions such as neurotransmitter recycling, synaptic control, and ionic equilibrium.^75^, ^76^, ^77^ In addition to glutamate‐induced excitotoxicity, glutamate‐induced excitotoxicity leads to PSP pathology by adjusting Ca^2+^ and K^+^ equilibrium, influencing the death of dopaminergic neurons.^78^, ^79^ Characteristic pathological features of PSP consist of globose and neurofibrillary tangles, tufted astrocytes, and twisted bodies.^80^ Recent studies have been advancing astrocyte therapy products for neurodegenerative diseases, including PSP.^81^ In a similar area, our findings revealed a strong correlation between excitatory and inhibitory neurons and the unusual gene expression contributing to reduced MSN in PSP. Earlier research indicated that equilibrium in the CNS is maintained by an excitatory/inhibitory (E/I) balance, and a disparity in E/I involving neurotransmitters such as DA, glutamate, GABA, acetylcholine, and serotonin plays a role in the disease progression of PSP.^82^, ^83^ Reduced DA in the substantia nigra pars compacta (SNc), leads to decreased GABAergic interneuron activity and simultaneously slows SNc transmission to the dorsal striatum, influencing PSP pathophysiology.^84^ Recent advances may lead to the development of treatments focused on both GABAergic and dopaminergic systems as innovative approaches for treating PSP.^85^

Finally, we associated changes in PET neurotransmitter receptor density maps with MSN differences in PSP, revealing correlations between seven receptors and MSN changes, while 5‐HT1A, 5‐HT1B, and 5‐HT4 are involved in various physiological processes such as emotion, cognition, and motor control.^86^, ^87^, ^88^, ^89^, ^90^, ^91^, ^92^, ^93^ D2 receptors are primarily associated with the pathogenesis of Parkinson's syndrome, characterized by the degeneration and death of dopamine neurons in the SNc, leading to significantly reduced dopamine neurotransmitter secretion in the striatum.^94^, ^95^, ^96^, ^97^, ^98^ The NET receptor is an important neurotransmitter transport protein that primarily regulates the transport and clearance of norepinephrine between neurons. The NET receptor modulates neurotransmitter concentrations in the synaptic cleft by reuptaking neurotransmitters, thereby influencing neural signal transmission.^99^, ^100^, ^101^, ^102^ Activation of α4β2 receptors can increase dopamine release, producing rewarding and cognitive enhancement effects.^103^, ^104^ The MOR receptor is an important type of opioid receptor that is primarily distributed in the central and peripheral nervous systems. Activating MOR receptors can induce analgesia, sedation, euphoria, and addictive effects.^105^

Several limitations of this study should be highlighted. First, the number of participants in this study was relatively small, and future studies with large sample sizes are needed to validate MSN abnormalities in PSP patients. Second, our research utilized available AHBA gene information gathered from the deceased brains of six donors who had no past neurological illnesses. Gene expression patterns derived from standard brain tissue could hinder investigations of connections between transcriptional data and large‐scale MSN abnormalities among different groups, rendering the study of unique patient impacts impractical. Third, our study exclusively utilized data from the left hemisphere because the AHBA dataset contains only two data from the right hemisphere. Consequently, the genetic activity linked to regional variations in MSN fails to mirror the comprehensive transcriptional data of the brain. In the future, enhancing sample collection from the complete brain of patients with postmortem PSP is crucial for discerning cerebral variations and improving our comprehension of regional selective susceptibility in PSP. Finally, the variability in the human brain's structure, capability, and behavior is due to multiple elements such as genetic differences, environmental exposure, and their interplay. In addition to genetics, the influence of the environment is vital in the development of neuropsychiatric conditions. Our research revealed transcriptional patterns associated with brain morphology, yet the specific environmental factors influencing these phenotypes are still ambiguous.^106^

## 5 CONSLUSION

In conclusion, our research revealed variations in brain cortex patterns in PSP patients, suggesting a primary reduction in MSN in the frontal and temporal cortex regions, and an increase in MSN in the occipital cortical area. By merging spatial morphometric data with transcriptional markers, it was ascertained that genes related to MSN play a role in astrocytes, excitatory, and inhibitory neurons, and significantly increase the number of neuron‐targeted biological mechanisms. The results have enhanced our comprehension of the minute genetic and cellular mechanisms responsible for large‐scale structural disorders, offering possible focal points for upcoming therapeutic studies.

## AUTHOR CONTRIBUTIONS

Concept and design: Junyu Qu; data collection and analysis: Junyu Qu, Yancai Qu, Rui Zhu, Yongsheng Wu and Guihua Xu; drafting of the article: Junyu Qu; critical revision of the article for important intellectual content: Dawei Wang; study supervision: Dawei Wang. All the authors approved the final article.

## CONFLICT OF INTEREST STATEMENT

The authors declare no conflict of interest.

## Supporting information


Appendix S1

## Data Availability

The neuroimaging preprocessing software is freely available (FreeSurfer v6.0, http://surfer.nmr.mgh.harvard.edu/, and FSL v6.0, https://fsl.fmrib.ox.ac.uk/fsl/fslwiki). The codes for MSN analysis and PLS are openly available at https://github.com/SarahMorgan/Morphometric_Similarity_SZ. The codes for gene expression analysis can be found at https://github.com/rmarkello/abagen. Gene enrichments were analyzed at https://metascape.org/gp/index.html#/main/step1. The code for spatial permutation testing can be found at https://github.com/frantisekvasa/rotate_parcellation. PET receptors can be found at https://github.com/netneurolab/hansen_receptors.
