# Supplementary material for: Transcriptional expression patterns of the cortical morphometric similarity network in progressive supranuclear palsy
Source: CNS Neurosci Ther. 2024 Aug 4;30(8):e14901. doi: 10.1111/cns.14901 (PMC11298202; doi:10.1111/cns.14901)
Supplement: Supplementary file 1 — Appendix S1 [file CNS-30-e14901-s001.docx]

**Supplementary Information**

Contents

**[Supplementary Text](#_Toc836)** [2](#_Toc836)

[1. Details of neuroimaging data acquisition 2](#_Toc21425)

[1.1 Discovery cohort 2](#_Toc23516)

[1.2 Replication cohort 3](#_Toc32691)

[2. Details of neuroimaging data preprocessing 3](#_Toc9493)

[3. Details of gene expression data preprocessing 4](#_Toc28781)

[4. Yeo functional networks and von Economo classes 6](#_Toc20825)

[5. Details of transcription-neuroimaging association analysis 7](#_Toc17768)

[6. Associations between the MSN scores and clinical variables in the PSP group 8](#_Toc17842)

[7. Details of correlations between MSN changes and PET of neurotransmitter receptors and transporters 8](#_Toc3236)

[8. Details of longitudinal MSN changes in PSP 9](#_Toc2254)

[9. Details of enrichment pathways of genes 10](#_Toc26618)

[10. Details of PSP-related genes from the previous GWAS 10](#_Toc15207)

[11. Details of cell–type specific expression 11](#_Toc27260)

[13. Validation: the TIV effect on case-control differences 13](#_Toc11417)

[14. Validation: details of spatial similarity analysis in the replication cohort 13](#_Toc21984)

[15. Validation: details of a meta-analysis of multiple gene lists 13](#_Toc12838)

**[Reference](#_Toc24632)** [15](#_Toc24632)

**[Supplementary Tables](#_Toc27588)** [17](#_Toc27588)

[Table S1. Demographic and clinical characteristics of the finally included subjects in discovery cohort. 17](#_Toc11403)

[Table S2. Demographic and clinical characteristics of the finally included subjects in replication cohort. 18](#_Toc4577)

[Table S3. Regional differences between PSP and healthy controls. 19](#_Toc13577)

[Table S4. Regional differences of PSP in longitudinal study. 20](#_Toc13190)

[Table S5. The differences in the MSN in each Yeo network. 21](#_Toc7140)

[Table S6. The differences in the MSN in each von Economo class. 22](#_Toc9272)

[Table S7. The relationship between the MSN of abnormal regions and the PSPRS and its subitems scores. 23](#_Toc16422)

[Table S8. PSP-related genes from the AHBA database. 26](#_Toc8717)

[Table S9. The top significant categories from Metascape based on the PLS1- genes using ensemble-based null models in GCEA. 27](#_Toc7669)

**[Supplementary Figures](#_Toc15700)** [28](#_Toc15700)

[Figure S1. TIV effect on case-control differences. 28](#_Toc32286)

[Figure S2. Longitudinal Study of Morphological Similarity Network Changes. 29](#_Toc5754)

[Figure S3. The significant correlations between the other five PSP-related genes from the AHBA database and the case-control t-map. 30](#_Toc22246)

[Figure S4. Enrichment pathways of PLS1+ gene list. 31](#_Toc27667)

[Figure S5. Shared pathways between the MSN differences-related genes and genes from GWAS. 32](#_Toc4205)

[Figure S6. Replicable regional MSN differences of PSP. 33](#_Toc558)

[Figure S7. Reproducibility analysis of the transcriptional enrichment of changes in MSN. 34](#_Toc12588)

#

# Supplementary Text

## 1. Details of neuroimaging data acquisition

### 1.1 Discovery cohort

MRI data gathering was executed through a 3T Siemens Tim Trio system, featuring a 12-channel head coil, at the University of California (UCSF) and Massachusetts General Hospital (MGH). The acquisition of T1-weighted images employed a volumetric magnetic rapid gradient-echo sequence (MPRAGE), characterized by repetition time (TR), echo time (TE), and inversion time (TI) values of 2300/2.98/900 ms, flip angle setting at 9°, slice thickness at 1 mm, a matrix dimension of 256 × 240 × 160, voxel dimensions of 1 × 1 × 1 mm^3^, and 160 sagittal sections. The capturing of resting-state functional images was achieved through a single-shot echo-planar-imaging (SS-EPI) sequence, which includes TR/TE ratios of 2000/27 ms, a matrix dimension of 92 × 92, an 80° flip angle, a thickness of 3 mm slices, voxel dimensions of 3 × 3 × 3 mm^3^, including 36 transverse slices and a total of 240 volumes. The diffusion tensor pictures were captured with a 2D SS-EPI sequence, consisting of this set: TR/TE ratio = 8200/86 ms, a flip angle of 180°, a slice thickness of 2.2 mm, a matrix dimension of 100 × 100, 65 cross-sectional slices, 64 noncollinear angles (b = 2000 s/mm^2^), and 1 b0 image (b = 0 s/mm^2^).

### **1.2 Replication cohort**

MRI occurred at Jinan's Qilu Hospital, China, employing an eight-channel head coil and a Siemens verio 3.0 Tesla MRI scanner. To reduce head motion, tight and tight foam padding was employed. Every participant was fitted with earplugs to aid in lowering scanner noise. Echo Gradient Data through resting-state functional magnetic resonance imaging (fMRI) was acquired utilizing the GRE-SS-EPI Single-Shot Echo-Planar Imaging sequence, applying these specific imaging parameters: a flip angle of 90°, a field dimensions of 220 mm × 220 mm, a matrix of 64 × 64, a repetition duration/echo interval of 2,000/30 ms, 36 lateral slices, and 180 volumes. Throughout the fMRI procedures, participants were directed to shut their eyes, stay as still as possible, disregard specific areas, and refrain from sleeping. The sagittal 3D T1-weighted scans were captured via a rapid acquisition gradient echo (MPRAGE) sequence prepared for magnetization (with repetition times/echo times = 2,000/2.3 ms; inversion time = 900 ms; flip angle = 9°; matrix of 256 × 256; slice thickness of 1 mm, no gap; 192 slices).

## Details of neuroimaging data preprocessing

For every participant, the brain's cortical region was reassembled using a 3D T1-weighted, high-resolution image in their unique environment, employing the “recon-all” process within FreeSurfer software (Version 7.3, accessible at <http://surfer.nmr.mgh.harvard.edu).> The procedures for preprocessing neuroimaging are delineated into five phases: (i) skull stripping involving the extraction of nonbrain tissues. To create images normalized for intensity, the irregularity in intensity was addressed; (ii) dividing tissue, which established the demarcation between gray (GM) and white matter (WM); (iii) reconstructing surfaces: constructing a bi-dimensional lattice to trace the WM-GM interface, thereby creating the WM surface across each hemisphere and expanding it outwardly to form the pial layer. Addressing topological flaws involved correcting the topology; (iv) reconstructing metrics on the surface, such as cortical thickness, surface area, volume, and curvature, derived from the pial and lighter matter surfaces, and (v) calculating parameters for spherical normalization: these were subsequently expanded into a spherical format and standardized against the fsaverage template to ascertain the spherical normalization figures. In addition, The DWI images were preprocessed on volumetric space using FMRIB Software Library (FSL, v6.0, <https://fsl.fmrib.ox.ac.uk/fsl/fslwiki).> Diffusion tensor models were then estimated using linear least squares fitting.

## Details of gene expression data preprocessing

The initial treatment of gene expression data is outlined in these steps: (i) updating probe-to-gene labels: replacing microarray probes with data from Arnatkevičiūtė et al. ^1^, removing unreliable probes failing to align with genes; (ii) filtering based on intensity: adhering to the earlier outlined method [1], probes with annotations limited to no more than background noise in half the tissue samples; (iii) choice of probes: a sole microarray marker with the greatest differential stability was picked for each gene ^2^, preserving 15,633 probes for 15,633 genes; (iv) aligning samples across different regions: assigning them to brain areas using Montreal Neurological Institute (MNI) coordinates, selecting the nearest areas within a 2 mm Euclidean range. Addressing the issue of misassignment bias involved limiting the alignment of samples from region to region through the division of hemisphere, cortical, and subcortical; also, when dealing with absent data, if a specific brain region was not allotted a sample as per the previously cited method, the gene expressions from the void regions were deduced by correlating the expression of the closest tissues samples. We utilized the averaged inverse distance of the closely plotted map to depict region's segmented expression levels, guaranteeing values were allocated to all; in case of sample normalization, a robustly scaled sigmoid curve normalized gene expression values of tissue samples for each donor, addressing variations between individuals. Subsequently, adjusted expression levels were adjusted to the standard unit interval; (vii) gene normalization involved normalizing gene expression values through identical tissue samples; (viii) metric for combining samples per region: averages of samples from the same brain area were calculated individually for each donor. Averaging scaled regional expression profiles among donors yielded a matrix aligning with brain areas and columns for the maintained 15,633 genes; (ix) gene stability selection process: a 0.1 threshold was set for gene stability differences to guarantee the retention of only the stable ones for additional analysis ^2,3^, and ultimately, a mere 15,632 genes remained. Owing to the fact that just two out of the six brains in the AHBA database contained samples from the right side, this research specifically focused on the left side. Consequently, for the following analyses, the gene expression matrix encompassing 152 regions × 15,632 genes was employed.

## Yeo functional networks and von Economo classes

To frame the key regional differences in MSN case-control, we consulted two previous cortical area classifications: the Yeo atlas, which is sorted by resting-state networks from functional MRI ^4^, and the von Economo atlas, which is categorized using cytoarchitectonic standards ^5^. Pursuing this, we initially determined the average MSN score across all areas in a specific Yeo network or von Economo class, and utilized General Linear Model (GLM) to delve into case-control variances in the MSN by excluding variables like age, gender, and age × sex.. Subsequently, we acquired the t-statistics and their respective p-values for each network-associated alteration in the MSN in a specific Yeo network (see Table S5) and for the von Economo class (see Table S6). Our findings indicate that in the Yeo functional networks atlas study, individuals with PSP exhibited a heightened MSN within their visual network (adjusted p = 4.33 × 10^-4^, BH-FDR corrected). In the von Economo cytoarchitectural atlas, PSP patients showed reduced MSN in the associate cortex 1 cytoarchitectural category (adjusted p = 8.30 × 10^-4^, BH-FDR corrected).

## Details of transcription-neuroimaging association analysis

PLS regression analysis ^6^ was executed to explore the connection between the expression levels of 15,632 genes and MSN changes between case and control (t values from 152 cortical zones in the left hemisphere). Within the PLS regression framework, every segment of the z-score normalized gene expression matrix (152 regions × 15,632 genes) served as the independent variable, with the z-score normalized MSN case-control t vector (152 regions × 1) functioning as the dependent variable. PLS elements, formed from a linear amalgamation of weighted gene expression numbers, are ordered according to the elucidated differences between independent and dependent variables. Consequently, the first PLS element (PLS1) serves as the most effective low-dimensional depiction of the covariance in high-dimensional data matrices ^7^. Investigating if the variance in the PLS element exceeded random expectations, a permutation test for space autocorrelation preservation was employed, known as the spin test ^8^, occurring 10,000 times. Furthermore, a bootstrapping approach was utilized to assess the importance of genes involved in various components. Precisely, post acquiring authentic PLS weights for every gene and element, we performed a 10,000-fold resampling, replacing 152 cortical areas, to refresh the PLS analysis and produced the respective weights for each gene. Using bootstrapping, we evaluated standard errors, and the z scores (calculated from PLS weights divided by their standard errors) were transformed into p values to determine the role each gene plays in each component of PLS. Solely crucial genes (BH-FDR adjusted p < 0.01) were preserved for later analytical examinations.

## Associations between the MSN scores and clinical variables in the PSP group

Our study focused on examining the links between modified MSN scores and various clinical indicators, such as the PSPRS rating along with its parts ^9^. We extracted MSN scores from important areas in each patient, evaluating how changes in MSN scores and clinical factors in case-control were correlated through pearson correlation analysis. Findings are presented in Table S7.

## Details of correlations between MSN changes and PET of neurotransmitter receptors and transporters

Our research utilized whole-brain neurotransmitter receptor mappings derived from PET, encompassing 19 distinct neurotransmitter receptors, binding sites, and transporters related to nine varied neurotransmitter systems ^10^. This was applied to over 1,200 healthy individuals and can be accessed at <https://github.com/netneurolab/hansen_receptors.> Then, we ompute the correlation between PSP-related MSN changes and 19 PET neurotransmitter receptors.

## Details of longitudinal MSN changes in PSP

Utilizing the PSP longitudinal study, we studied the variations of MSN in PSP at three time points—baseline, six months, and one year (uncorrected p < 0.05). At six months, the MSN primarily decreased in the left fusiform (part 3), left inferior parietal (part 2), and left superior frontal (part 2) regions, and increased in the left caudal anterior cingulate (part 1), left parstriangularis (part 2), left posterior cingulate (part 1 and part 2), right caudal anterior cingulate (part 1) and right precuneus (part 3) regions; after one year, the changes in MSN predominantly occurred with an increase in the right lateral orbitofrontal (part 2) region, and a decrease in the left precuneus (part 4), right inferior parietal (part 9) and right supramarginal (part 3) regions; however, comparisons between six months and one year indicated that the changes in the MSN were mainly concentrated in the reduction in the left parstriangularis (part 1), right inferior parietal (part 6), right pericalcarine (part 2), right supramarginal (part 3) regions, and an increase in the left temporal pole (part 1), left insula (part 3), right superior frontal (part 13) and right temporal pole (part 1) regions (Figure S2 and Table S4).

## Details of enrichment pathways of genes

We employed Metascape for annotations of gene functions and utilized 15,632 genes, each with appropriate brain expression information, as the background. The enrichment studies showed a primary enrichment in the PLS1− genes within GO biological processes, including “neuron projection development,” “organic hydroxy compound metabolic process,” “synaptic signaling,” “response to toxic substance,” “regulation of secretion by cell,” “synapse organization,” and “cellular response to metal ion,” and with notable presence in Reactome gene sets, namely "Nervous system development" and "Neurotransmitter receptors and postsynaptic signal transmission" (p < 0.01; Figure 4A, B). In addition, PLS+ genes showed an enhancement in various biological activities such as “chromatin organization,” “DNA metabolic process,” “head development,” “RNA splicing,” and “intracellular receptor signaling pathway,” and were significantly enriched in two KEGG pathways, “MAPK signaling pathway” and “calcium signaling pathway”. (p < 0.01; Figure S4).

## Details of PSP-related genes from the previous GWAS

To explore identical enrichment pathways linking PLS1− genes to polygenic risk genes for PSP, a meta-analysis involving multiple genes between PLS1− genes and potential risk genes from PSP genome-wide association studies (GWAS) was conducted ^11-15^. The results suggest that the PLS1− genes aligned in the top 7 enrichment pathways identical to those in the GWAS, implying functional consistency with earlier research. The leading 7 pathways included “neuron projection development,” “axon,” “neurotransmitter receptors and postsynaptic signal transmission,” “dendrite,” “regulation of neuron projection development,” “behavior,” “circadian synchronization,” “secret secretion regulation,” “trans-synaptic signaling regulation,” and “regulation of synapse structure or activity” (p < 0.01; Figure S5).

## Details of cell–type specific expression

In pursuit of enhancing our study's accuracy and taking into account brain cellular diversity, we adopted a more nuanced method by categorizing PLS1− genes into seven primary cell types ^16^: astrocytes, microglia, oligodendrocyte precursors (OPCs), oligodendrocytes, endothelial cells, excitatory neurons and inhibitory neurons, pinpointing distinct cell types that are predisposed to MSN changes in our research. Within the PLS1− gene list, 777 genes showed significant involvement in astrocytes (p < 0.0001, FDR-corrected), 1133 genes played a major role in excitatory neurons (p < 0.0001, FDR-corrected), 831 genes had a notable contribution to inhibitory neurons (p < 0.0001, FDR-corrected), and 784 genes were significantly involved in oligodendrocytes (p< 0.0001, FDR-corrected) (Figure 4C, D).

Genetic enrichment studies focusing on specific cell types showed that alterations in the MSN in PSP patients exhibited a notable enrichment in reactome gene sets linked to signaling by receptor tyrosine kinases in astrocytes (Figure 4E). Alterations in MSN within neuronal cells were enriched in GO terms such as “response to hormone”, “cellular response to cytokine stimulus”, “cellular response to lipid”, and “leukocyte activation”. Overall, our method revealed alterations in gene expression linked to MSN in various cell types, enabling us to accurately identify particular cell types connected to PSP pathology.

1. **Details of relationship between MSN in PSP and neurotransmitter receptors**

By evaluating the influence of neuromodulatory systems on MSN variance in PSP, our research focused on the relationship between neurotransmitter receptor density patterns and MSN alterations in PSP patients, in contrast to HCs. Our findings indicated a notable correlation between MSN alterations in PSP and the regional variations in the density patterns of serotoninergic, dopaminergic, norepinephrine, acetylcholine, and opioid receptors (Figure 5, 5-HT1A receptor, r = -0.52, adjusted p = 2.5×10^-4^; 5-HT1B receptor, r = 0.36, adjusted p = 0.022; 5-HT4 receptor, r = -0.33, adjusted p = 0.033; D2 receptor, r = -0.31, adjusted p = 0.023; NET receptor, r = -0.48, adjusted p = 6.5×10^-4^; α4β2 receptor, r = 0.35, adjusted p = 0.0064; MOR receptor, r = -0.48, adjusted p = 9.5×10^-4^). The changes of MSN in PSP correlated specifically with a decrease in the D2 receptor, NET receptor, MOR receptor, and two serotonin receptors (5-HT1A and 5-HT4), as did increases in the levels of the α4β2 and 5-HT1B receptors.

## Validation: the TIV effect on case-control differences

Our study found that TIV remained consistently similar between PSP patients and the healthy subjects (two-sample t-test, t = 0.62, p = 0.54). Additionally, TIV was integrated as a covariate to confirm the strength of the existing findings. The findings indicated comparable case-control variances in theMSN when TIV was included as a covariate to the MSN scenario without TIV (Figure S5).

## Validation: details of spatial similarity analysis in the replication cohort

The previously mentioned variations in MSN case-control were confirmed through the replication cohort analysis. Utilizing the same methodology as in the discovery cohort, the case-control t-map was derived from the replication cohort, applying GLM to regression analysis of age, sex, and age × sex.. For verifying the reproducibility of MSN-related regional variations, an analysis of spatial likeness was carried out on t-value maps comparing discovery and replication groups ^17,18^. We found that the PSP-HC (case-control) t-map obtained from the duplicate group showed a spatial alignment to that of the discovery cohort (r = 0.52, p_spin_ < 0.0001; Figure S6).

## Validation: details of a meta-analysis of multiple gene lists

To authenticate the gene list linked to PSP acquired through MSN analysis, a multi-gene list meta-analysis was performed comparing the PLS1− gene lists of the research and duplicated groups. Each pathway acquired underwent a significance threshold of 5%, adjusted according to the FDR. The extent of gene overlap was assessed using the OR. Delving deeper into the confirmation of transcriptional enrichment in MSN alterations, a comprehensive meta-analysis involving multiple genes of PLS1− was conducted in both the discovery and replication groups. Our replication cohort revealed a significant overexpression of in 1579 PLS1+  (Z > 3) genes and 1129 PLS−  (Z < − 3) genes within cortical areas, comprising 2708 variations in regional MSN gene lists. There was a strong intersection in the gene lists of the discovery and replication groups: odds ratio (OR) = 1.46, p < 0.0001. Furthermore, our research revealed enrichment pathways shared between the discovery and replication groups characterized by a notable intersection of PLS1− genes: OR = 4.62, p < 0.0001 (Figure S7A). Intersecting ontologies used by the discovery and replication groups focused on terms such as “presynapse”, “neuron projection development”, “glutamatergic synapse”, “synaptic signaling”, “oxidoreductase activity”, “axon”. and “organic hydroxy compound metabolic process” (Figure S7B, C). A substantial overlap in ontology terms supports the broad correlation between gene expression and changes in MSN linked to PSP.

# Reference

1 Arnatkeviciute, A., Fulcher, B. D. & Fornito, A. A practical guide to linking brain-wide gene expression and neuroimaging data. *NeuroImage* **189**, 353-367, doi:10.1016/j.neuroimage.2019.01.011 (2019).

2 Hawrylycz, M. *et al.* Canonical genetic signatures of the adult human brain. *Nature Neuroscience* **18**, 1832-1844, doi:10.1038/nn.4171 (2015).

3 Hansen, J. Y. *et al.* Mapping gene transcription and neurocognition across human neocortex. *Nature Human Behaviour* **5**, 1240-1250, doi:10.1038/s41562-021-01082-z (2021).

4 Thomas Yeo, B. T. *et al.* The organization of the human cerebral cortex estimated by intrinsic functional connectivity. *Journal of Neurophysiology* **106**, 1125-1165, doi:10.1152/jn.00338.2011 (2011).

5 Economo, C. F. v., Koskinas, G. N. & Triarhou, L. C.

6 Abdi, H. Partial least squares regression and projection on latent structure regression (PLS Regression). *WIREs Computational Statistics* **2**, 97-106, doi:<https://doi.org/10.1002/wics.51> (2010).

7 Abdi, H. & Williams, L. J. Partial least squares methods: partial least squares correlation and partial least square regression. *Methods Mol Biol* **930**, 549-579, doi:10.1007/978-1-62703-059-5_23 (2013).

8 Váša, F. *et al.* Adolescent Tuning of Association Cortex in Human Structural Brain Networks. *Cerebral Cortex* **28**, 281-294, doi:10.1093/cercor/bhx249 (2018).

9 Höglinger, G. U. *et al.* Clinical diagnosis of progressive supranuclear palsy: The movement disorder society criteria. *Movement Disorders* **32**, 853-864, doi:10.1002/mds.26987 (2017).

10 Hansen, J. Y. *et al.* Mapping neurotransmitter systems to the structural and functional organization of the human neocortex. *Nature Neuroscience* **25**, 1569-1581, doi:10.1038/s41593-022-01186-3 (2022).

11 Hoglinger, G. U. *et al.* Identification of common variants influencing risk of the tauopathy progressive supranuclear palsy. *Nat Genet* **43**, 699-705, doi:10.1038/ng.859 (2011).

12 Kouri, N. *et al.* Latent trait modeling of tau neuropathology in progressive supranuclear palsy. *Acta Neuropathologica* **141**, 667-680, doi:10.1007/s00401-021-02289-0 (2021).

13 Jabbari, E. *et al.* Genetic determinants of survival in progressive supranuclear palsy: a genome-wide association study. *The Lancet Neurology* **20**, 107-116, doi:10.1016/s1474-4422(20)30394-x (2021).

14 Chen, J. A. *et al.* Joint genome-wide association study of progressive supranuclear palsy identifies novel susceptibility loci and genetic correlation to neurodegenerative diseases. *Molecular Neurodegeneration* **13**, doi:10.1186/s13024-018-0270-8 (2018).

15 Sanchez-Contreras, M. Y. *et al.* Replication of progressive supranuclear palsy genome-wide association study identifies SLCO1A2 and DUSP10 as new susceptibility loci. *Molecular Neurodegeneration* **13**, doi:10.1186/s13024-018-0267-3 (2018).

16 Seidlitz, J. *et al.* Transcriptomic and cellular decoding of regional brain vulnerability to neurogenetic disorders. *Nature Communications* **11**, 3358, doi:10.1038/s41467-020-17051-5 (2020).

17 Morgan, S. E. *et al.* Cortical patterning of abnormal morphometric similarity in psychosis is associated with brain expression of schizophrenia-related genes. *Proceedings of the National Academy of Sciences* **116**, 9604-9609, doi:10.1073/pnas.1820754116 (2019).

18 Holiga, Š. *et al.* Patients with autism spectrum disorders display reproducible functional connectivity alterations. **11**, eaat9223, doi:doi:10.1126/scitranslmed.aat9223 (2019).

# Supplementary Tables

## Table S1. Demographic and clinical characteristics of the finally included subjects in discovery cohort.

| Variables | PSP at baseline  (*n* = 47) | PSP in 6 months  (*n* = 31) | PSP in 1 year  (*n* = 16) | HC  (*n* = 80) | *p* value |
| --- | --- | --- | --- | --- | --- |
| Gender (M/F) | 22/25 | - | - | 31/49 | 0.37^a^ |
| Age (years) | 70.04±7.46 | - | - | 67.73±6.35 | 0.08^b^ |
| Duration (years) | 5.68±4.05 | - | - | - | - |
| Education | 15.89±4.28 | - | - | - | - |
| PSPRS | 36.97±16.92 | - | - | - | - |
| History | 8.26±4.18 | - | - | - | - |
| Mentation | 3.69±2.49 | - | - | - | - |
| Bulbar | 2.79±1.75 | - | - | - | - |
| Ocular motor | 7.61±4.10 | - | - | - | - |
| Limb motor | 4.79±2.40 | - | - | - | - |
| Gait and midline | 9.82±5.56 | - | - | - | - |

^a^ The *p* value is obtained by Chi-square test.

^b^ The *p* value is obtained by two-sample *t*-test.

Data of the continuous variables are shown as mean±SD.

Abbreviations: M, male; F, female; PSP, progressive supranuclear palsy; HC, healthy controls; PSPRS, Progressive Supranuclear Palsy Rating Scale.

## Table S2. Demographic and clinical characteristics of the finally included subjects in replication cohort.

| Variables | PSP (*n* = 51) | HC (*n* =54) | *p* value |
| --- | --- | --- | --- |
| Gender (M/F) | 28/23 | 20/34 | 0.07^a^ |
| Age (years) | 64.45±6.50 | 64.22±4.47 | 0.83^b^ |
| Duration (years) | 3.67±1.69 | - | - |
| Education | 9.19±4.44 | - | - |
| PSPRS | 37.42±15.86 | - | - |
| History | 8.79±4.72 | - | - |
| Mentation | 2.44±2.91 | - | - |
| Bulbar | 3.30±1.67 | - | - |
| Ocular motor | 7.21±4.07 | - | - |
| Limb motor | 4.93±3.45 | - | - |
| Gait and midline | 10.74±3.87 | - | - |

^a^ The *p* value is obtained by Chi-square test.

^b^ The *p* value is obtained by two-sample *t*-test.

Data of the continuous variables are shown as mean±SD.

Abbreviations: M, male; F, female; PSP, progressive supranuclear palsy; HC, healthy controls; PSPRS, Progressive Supranuclear Palsy Rating Scale.

## Table S3. Regional differences between PSP and healthy controls.

| Regions | MNI coordinates (x, y, z) | | | *t*-statistic | *p* value |
| --- | --- | --- | --- | --- | --- |
| Lh fusiform part1 | -30.24 | -46.49 | -17.45 | -2.97 | 3.58×10^-3^ |
| Lh lateraloccipital part3 | -14.74 | -99.83 | 8.84 | 4.04 | 9.35×10^-5^ |
| Lh lateraloccipital part5 | -16.53 | -100.15 | -6.05 | 3.09 | 2.50×10^-3^ |
| Lh lateraloccipital part7 | -28.70 | -89.41 | 2.44 | 3.00 | 3.27×10^-3^ |
| Lh lateralorbitofrontal part4 | -28.86 | 25.38 | -12.55 | -2.99 | 3.37×10^-3^ |
| Lh lingual part4 | -6.98 | -76.65 | -1.66 | 3.15 | 2.05×10^-3^ |
| Lh lingual part6 | -15.83 | -74.54 | -9.20 | 3.90 | 1.57×10^-4^ |
| Lh parahippocampal part1 | -25.99 | -25.19 | -25.33 | -3.72 | 3.02×10^-4^ |
| Lh postcentral part4 | -60.99 | -13.47 | 23.99 | 2.85 | 5.13×10^-3^ |
| Lh superiorfrontal part2 | -11.69 | -8.42 | 64.79 | -3.42 | 8.50×10^-4^ |
| Lh superiorfrontal part4 | -17.93 | 1.24 | 62.20 | -3.06 | 2.72×10^-3^ |
| Lh superiortemporal part2 | -43.70 | 8.44 | -23.51 | -3.43 | 8.22×10^-4^ |
| Lh superiortemporal part5 | -59.11 | -24.14 | -1.90 | -3.74 | 2.81×10^-4^ |
| Lh superiortemporal part6 | -55.63 | -12.86 | -2.43 | -2.97 | 3.58×10^-3^ |
| Lh superiortemporal part7 | -51.98 | -7.21 | -8.81 | -3.89 | 1.63×10^-4^ |
| Rh cuneus part2 | 6.20 | -89.29 | 13.00 | 3.65 | 3.87×10^-4^ |
| Rh cuneus part3 | 8.63 | -72.20 | 16.80 | 2.84 | 5.28×10^-3^ |
| Rh fusiform part3 | 37.49 | -59.46 | -16.18 | -3.72 | 3.02×10^-4^ |
| Rh fusiform part5 | 33.58 | -48.04 | -16.66 | -3.47 | 7.18×10^-4^ |
| Rh lateraloccipital part1 | 18.65 | -99.16 | -7.39 | 3.09 | 2.48×10^-3^ |
| Rh lateraloccipital part3 | 17.11 | -99.07 | 10.07 | 3.16 | 1.99×10^-3^ |
| Rh lateraloccipital part6 | 32.22 | -84.39 | 8.26 | 3.91 | 1.52×10^-4^ |
| Rh lateraloccipital part7 | 28.68 | -86.71 | -12.20 | 2.84 | 5.28×10^-3^ |
| Rh lingual part2 | 8.20 | -89.14 | -8.57 | 3.17 | 1.92×10^-3^ |
| Rh lingual part4 | 7.69 | -77.24 | -3.47 | 3.54 | 5.66×10^-4^ |
| Rh lingual part5 | 12.89 | -63.72 | 2.18 | 3.84 | 1.96×10^-4^ |
| Rh lingual part6 | 17.12 | -73.04 | -8.47 | 4.72 | 6.30×10^-6^ |
| Rh pericalcarine part1 | 14.57 | -70.59 | 8.20 | 3.59 | 4.76×10^-4^ |
| Rh pericalcarine part3 | 9.32 | -78.77 | 7.68 | 2.89 | 4.56×10^-3^ |
| Rh superiortemporal part2 | 60.40 | -31.75 | 9.37 | -3.01 | 3.17×10^-3^ |
| Rh superiortemporal part5 | 59.15 | -22.56 | 3.01 | -3.05 | 2.80×10^-3^ |
| Rh superiortemporal part6 | 51.44 | -17.54 | -3.44 | -3.20 | 1.75×10^-3^ |
| Rh insula part4 | 36.77 | 5.57 | -1.36 | -3.32 | 1.19×10^-3^ |

Note: A GLM was used to investigate regionally SFC alterations in PSP group, while regressing out the effect of age, sex and age × sex. The *t*-statistic > 0 means PSP > HC, and the *t*-statistic < 0 means PSP < HC. All *p*-values survived after BH-FDR correction with *p* < 0.05. Abbreviations: BH-FDR, Benjamini-Hochberg false discovery rate; GLM, general linear model; PSP, progressive supranuclear palsy; HC: healthy controls.

## Table S4. Regional differences of PSP in longitudinal study.

| Regions | MNI coordinates (x, y, z) | | | *t*-statistic | *p* value |
| --- | --- | --- | --- | --- | --- |
| **Differences between 6months and baseline (p < 0.05 without FDR correction)** | | | | | |
| Lh caudalanteriorcingulate part1 | -5.00 | 20.65 | 25.73 | 3.71 | 8.42×10^-4^ |
| Lh fusiform part3 | -33.52 | -72.22 | -14.26 | -2.11 | 4.33×10^-2^ |
| Lh inferiorparietal part2 | -35.98 | -83.13 | 18.93 | -2.56 | 1.57×10^-2^ |
| Lh parstriangularis part2 | -40.49 | 31.11 | -0.50 | 2.31 | 2.79×10^-2^ |
| Lh posteriorcingulate part1 | -6.45 | -26.23 | 38.55 | 2.11 | 4.33×10^-2^ |
| Lh posteriorcingulate part2 | -4.38 | -7.35 | 35.44 | 2.13 | 4.15×10^-2^ |
| Lh superiorfrontal part2 | -11.69 | -8.42 | 64.79 | -2.35 | 2.55×10^-2^ |
| Rh caudalanteriorcingulate part1 | 5.64 | 21.08 | 26.73 | 2.38 | 2.39×10^-2^ |
| Rh precuneus part3 | 9.76 | -47.66 | 59.44 | 2.37 | 2.44×10^-2^ |
| **Differences between 1 year and baseline (p < 0.05 without FDR correction)** | | | | | |
| Lh precuneus part4 | -9.26 | -44.10 | 43.36 | -2.53 | 2.31×10^-2^ |
| Rh inferiorparietal part9 | 38.15 | -64.76 | 44.78 | -2.18 | 4.56×10^-2^ |
| Rh lateralorbitofrontal part2 | 18.68 | 45.83 | -18.56 | 2.08 | 5.51×10^-2^ |
| Rh supramarginal part3 | 52.15 | -41.40 | 41.96 | -2.47 | 2.60×10^-2^ |
| **Differences between 1 year and 6 months (p < 0.01 without FDR correction)** | | | | | |
| Lh parstriangularis part1 | -48.10 | 30.82 | 5.23 | -3.66 | 2.32×10^-3^ |
| Lh temporalpole part1 | -31.54 | 10.66 | -35.59 | 3.70 | 2.14×10^-3^ |
| Lh insula part3 | -37.40 | -8.59 | 4.44 | 3.22 | 5.72×10^-3^ |
| Rh inferiorparietal part6 | 42.33 | -50.82 | 40.64 | -4.00 | 1.16×10^-3^ |
| Rh pericalcarine part2 | 12.27 | -91.43 | 3.47 | -3.04 | 8.27×10^-3^ |
| Rh superiorfrontal part13 | 9.66 | 30.31 | 45.21 | 3.95 | 1.28×10^-3^ |
| Rh supramarginal part3 | 52.15 | -41.40 | 41.96 | -3.36 | 4.30×10^-3^ |
| Rh temporalpole part1 | 32.33 | 9.84 | -32.33 | 3.11 | 7.17×10^-3^ |

Note: A GLM was used to investigate regionally MSN alterations in PSP group in longitudinal study, Abbreviations: FDR, False discovery rate; GLM, general linear model; PSP, progressive supranuclear palsy; HC: healthy controls.

## Table S5. The differences in the MSN in each Yeo network.

| Statistics | VN | SMN | DAN | VAN | LN | FPN | DMN |
| --- | --- | --- | --- | --- | --- | --- | --- |
| *t*-statistic | 3.618 | -0.265 | -0.316 | -0.921 | -1.389 | 0.704 | -2.569 |
| *p*-value | 4.33×10^-4*^ | 0.7918 | 0.7525 | 0.3588 | 0.1674 | 0.4830 | 0.0114* |

Note: A GLM was used to investigate regionally MSN alterations in PSP group, while regressing out the effect of age, sex and age × sex. The *t*-statistic > 0 means PSP > HC, and the *t*-statistic < 0 means PSP < HC. * indicates that the *p* value was survived after BH-FDR correction. Abbreviations: BH-FDR, Benjamini-Hochberg false discovery rate; DAN, dorsal attention network; DMN, default mode network; FPN, fronto-parietal network; GLM, general linear model; LN, limbic network; MS, morphometric similarity; SMN, somato-motor network; VAN, ventral attention network; VN, visual network; MSN, Morphometric Similarity Network.

## Table S6. The differences in the MSN in each von Economo class.

| Statistics | Prim motor | Asso 1 | Asso 2 | Sec sens | Prim sens | Limbic | Insula |
| --- | --- | --- | --- | --- | --- | --- | --- |
| *t*-statistic | -1.249 | -3.428 | 0.694 | 2.659 | 1.849 | -0.546 | -1.710 |
| *p*-value | 0.214 | 8.30×10^-4*^ | 0.4893 | 0.0089* | 0.0669 | 0.5857 | 0.0897 |

Note: A GLM was used to investigate regionally MSN alterations in PSP group, while regressing out the effect of age, sex and age × sex. The *t*-statistic > 0 means PSP > HC, and the *t*-statistic < 0 means PSP < HC. * indicates that the *p* value was survived after BH-FDR correction. Abbreviations: Asso1, association cortex1; Asso2, association cortex2; BH-FDR, Benjamini-Hochberg false discovery rate; GLM, general linear model; Limbic, limbic regions; Insula, insular cortex; Prim motor, primary motor; Prim sens, primary sensory cortex; Sec sens, second sensory cortex; MSN, Morphometric Similarity Network.

## Table S7. The relationship between the MSN of abnormal regions and the PSPRS and its subitems scores.

| Regions | PSPRS | | History | | Mentation | | Bulbar | | Ocular motor | | Limb motor | | Gait and midline | |
| --- | --- | --- | --- | --- | --- | --- | --- | --- | --- | --- | --- | --- | --- | --- |
|  | *r value* | *p value* | *r value* | *p value* | *r value* | *p value* | *r value* | *p value* | *r value* | *p value* | *r value* | *p value* | *r value* | *p value* |
| Lh fusiform part1 | 0.120 | 0.421 | 0.174 | 0.241 | 0.088 | 0.557 | 0.081 | 0.589 | 0.146 | 0.328 | 0.086 | 0.564 | 0.051 | 0.732 |
| Lh lateraloccipital part3 | 0.030 | 0.842 | 0.042 | 0.782 | 0.167 | 0.263 | 0.052 | 0.726 | -0.114 | 0.446 | 0.046 | 0.757 | 0.045 | 0.766 |
| Lh lateraloccipital part5 | 0.059 | 0.693 | 0.054 | 0.720 | 0.139 | 0.353 | 0.015 | 0.921 | 0.003 | 0.982 | 0.078 | 0.601 | 0.053 | 0.725 |
| Lh lateraloccipital part7 | 0.119 | 0.427 | 0.079 | 0.597 | 0.181 | 0.223 | 0.052 | 0.729 | 0.037 | 0.807 | 0.137 | 0.358 | 0.149 | 0.317 |
| Lh lateralorbitofrontal part4 | 0.051 | 0.733 | 0.097 | 0.517 | -0.028 | 0.854 | -0.013 | 0.932 | 0.157 | 0.293 | -0.016 | 0.912 | -0.001 | 0.994 |
| Lh lingual part4 | 0.114 | 0.444 | 0.040 | 0.788 | 0.172 | 0.248 | 0.156 | 0.295 | 0.094 | 0.529 | 0.097 | 0.516 | 0.112 | 0.452 |
| Lh lingual part6 | 0.086 | 0.565 | 0.040 | 0.787 | 0.054 | 0.719 | 0.064 | 0.670 | 0.168 | 0.258 | 0.068 | 0.649 | 0.054 | 0.716 |
| Lh parahippocampal part1 | -0.063 | 0.672 | -0.126 | 0.399 | -0.160 | 0.283 | -0.044 | 0.769 | 0.149 | 0.318 | -0.107 | 0.474 | -0.093 | 0.533 |
| Lh postcentral part4 | 0.223 | 0.133 | 0.264 | 0.073 | 0.193 | 0.193 | 0.114 | 0.447 | 0.091 | 0.543 | 0.225 | 0.128 | 0.244 | 0.098 |
| Lh superiorfrontal part2 | 0.076 | 0.612 | 0.099 | 0.507 | 0.145 | 0.332 | 0.078 | 0.602 | -0.059 | 0.696 | 0.157 | 0.292 | 0.062 | 0.681 |
| Lh superiorfrontal part4 | 0.052 | 0.729 | 0.027 | 0.856 | -0.049 | 0.743 | 0.130 | 0.384 | 0.106 | 0.480 | 0.046 | 0.761 | 0.032 | 0.830 |
| Lh superiortemporal part2 | -0.165 | 0.268 | -0.192 | 0.197 | -0.131 | 0.381 | -0.146 | 0.328 | -0.071 | 0.634 | -0.156 | 0.296 | -0.173 | 0.246 |
| Lh superiortemporal part5 | 0.142 | 0.341 | 0.138 | 0.354 | -0.010 | 0.949 | 0.063 | 0.675 | 0.203 | 0.172 | 0.154 | 0.302 | 0.126 | 0.399 |
| Lh superiortemporal part6 | 0.042 | 0.777 | -0.010 | 0.948 | -0.009 | 0.953 | 0.108 | 0.470 | 0.201 | 0.175 | -0.020 | 0.892 | -0.023 | 0.881 |
| Lh superiortemporal part7 | -0.029 | 0.845 | -0.049 | 0.746 | -0.076 | 0.609 | 0.002 | 0.989 | 0.111 | 0.459 | -0.117 | 0.433 | -0.057 | 0.702 |
| Rh cuneus part2 | 0.124 | 0.407 | -0.007 | 0.962 | 0.205 | 0.168 | 0.188 | 0.207 | 0.108 | 0.470 | 0.082 | 0.583 | 0.154 | 0.302 |
| Rh cuneus part3 | 0.150 | 0.315 | 0.073 | 0.626 | 0.191 | 0.197 | 0.223 | 0.132 | 0.144 | 0.333 | 0.033 | 0.823 | 0.167 | 0.263 |
| Rh fusiform part3 | -0.063 | 0.676 | -0.028 | 0.854 | -0.073 | 0.624 | -0.108 | 0.471 | -0.021 | 0.889 | -0.030 | 0.840 | -0.093 | 0.536 |
| Rh fusiform part5 | -0.084 | 0.573 | -0.044 | 0.767 | -0.126 | 0.399 | -0.198 | 0.182 | 0.052 | 0.730 | -0.100 | 0.503 | -0.125 | 0.404 |
| Rh lateraloccipital part1 | 0.066 | 0.661 | 0.039 | 0.794 | 0.093 | 0.533 | 0.083 | 0.580 | 0.005 | 0.976 | 0.126 | 0.398 | 0.062 | 0.680 |
| Rh lateraloccipital part3 | 0.022 | 0.883 | 0.033 | 0.827 | 0.096 | 0.521 | 0.057 | 0.705 | -0.068 | 0.652 | 0.063 | 0.676 | 0.012 | 0.936 |
| Rh lateraloccipital part6 | 0.113 | 0.448 | 0.071 | 0.636 | 0.214 | 0.149 | 0.195 | 0.190 | 0.065 | 0.662 | 0.044 | 0.770 | 0.101 | 0.499 |
| Rh lateraloccipital part7 | 0.106 | 0.479 | 0.107 | 0.475 | 0.086 | 0.564 | 0.144 | 0.334 | 0.111 | 0.457 | 0.083 | 0.578 | 0.066 | 0.660 |
| Rh lingual part2 | 0.126 | 0.399 | 0.052 | 0.728 | 0.145 | 0.332 | 0.104 | 0.486 | 0.079 | 0.598 | 0.157 | 0.290 | 0.154 | 0.303 |
| Rh lingual part4 | 0.138 | 0.353 | 0.078 | 0.602 | 0.148 | 0.322 | 0.169 | 0.256 | 0.073 | 0.626 | 0.143 | 0.337 | 0.164 | 0.270 |
| Rh lingual part5 | 0.119 | 0.425 | 0.053 | 0.723 | 0.156 | 0.295 | 0.199 | 0.180 | 0.110 | 0.462 | 0.042 | 0.778 | 0.125 | 0.402 |
| Rh lingual part6 | 0.144 | 0.334 | 0.119 | 0.424 | 0.093 | 0.535 | 0.123 | 0.408 | 0.130 | 0.385 | 0.147 | 0.323 | 0.144 | 0.336 |
| Rh pericalcarine part1 | 0.061 | 0.684 | -0.008 | 0.955 | 0.086 | 0.565 | 0.153 | 0.303 | 0.052 | 0.731 | 0.016 | 0.914 | 0.079 | 0.596 |
| Rh pericalcarine part3 | 0.124 | 0.406 | 0.055 | 0.713 | 0.174 | 0.241 | 0.198 | 0.182 | 0.117 | 0.435 | 0.051 | 0.732 | 0.123 | 0.410 |
| Rh superiortemporal part2 | -0.078 | 0.602 | 0.000 | 1.000 | -0.180 | 0.225 | -0.158 | 0.288 | -0.013 | 0.931 | -0.068 | 0.648 | -0.093 | 0.532 |
| Rh superiortemporal part5 | -0.017 | 0.909 | 0.055 | 0.713 | -0.041 | 0.787 | -0.065 | 0.664 | 0.011 | 0.943 | -0.003 | 0.982 | -0.069 | 0.643 |
| Rh superiortemporal part6 | 0.029 | 0.847 | 0.045 | 0.763 | 0.005 | 0.974 | 0.038 | 0.797 | 0.209 | 0.159 | -0.070 | 0.638 | -0.078 | 0.603 |
| Rh insula part4 | -0.235 | 0.112 | -0.263 | 0.074 | -0.222 | 0.133 | -0.193 | 0.194 | -0.163 | 0.273 | -0.243 | 0.099 | -0.186 | 0.209 |

Note: The pearson correlation analysis was used to investigate the relationship between the SFC of abnormal regions and the clinical scores. * indicates that the *p* value was survived after BH-FDR correction. Abbreviations: BH-FDR, Benjamini-Hochberg false discovery rate; PSP, progressive supranuclear palsy; PSPRS, Progressive Supranuclear Palsy Rating Scale; MSN, Morphometric Similarity Network.

## Table S8. PSP-related genes from the AHBA database.

| Gene | Description | Gene Family |
| --- | --- | --- |
| **ATP1A3*** | ATPase, Na+/K+ transporting, alpha 3 polypeptide | transporter |
| DBH | dopamine beta-hydroxylase (dopamine beta-monooxygenase) | metabolic enzyme |
| DDC | dopa decarboxylase | metabolic enzyme |
| HTRA2 | HtrA serine peptidase 2 | metabolic enzyme |
| **LRRK2*** | leucine-rich repeat kinase 2 | kinase |
| **NR4A2*** | nuclear receptor subfamily 4, group A, member 2 | transcription factor |
| PARK2 | Parkinson disease (autosomal recessive, juvenile) 2, parkin | other intracellular |
| PARK7 | Parkinson disease (autosomal recessive, early onset) 7 | other intracellular |
| PINK1 | PTEN induced putative kinase 1 | kinase |
| SLC6A3 | solute carrier family 6 (neurotransmitter transporter, dopamine), member 3 | transporter |
| **SNCA*** | synuclein, alpha (non A4 component of amyloid precursor) | signal transduction |
| **SNCAIP*** | synuclein, alpha interacting protein (synphilin) | signal transduction |
| **SNCB*** | synuclein, beta | signal transduction |
| TH | tyrosine hydroxylase | metabolic enzyme |
| **UCHL1*** | ubiquitin carboxyl-terminal esterase L1 (ubiquitin thiolesterase) | other intracellular |

Note: Gene symbols in bold indicate these genes were contained in the list of 15,632 background genes. * indicates that these genes were significantly correlated with the case-control *t*-map (all *p_spin_s* < 0.05, BH-FDR corrected). Abbreviations: AHBA, Allen Human Brain Atlas; BH-FDR, Benjamini-Hochberg false discovery rate.

## Table S9. The top significant categories from Metascape based on the PLS1- genes using ensemble-based null models in GCEA.

| Categories | *p* value |
| --- | --- |
| presynapse | 1.94907E-19 |
| glutamatergic synapse | 2.77774E-17 |
| neuron projection development | 4.33126E-16 |
| axon | 1.74299E-15 |
| oxidoreductase activity | 5.31514E-15 |
| organic hydroxy compound metabolic process | 1.53168E-14 |
| synaptic signaling | 1.45992E-13 |
| response to toxic substance | 5.08818E-13 |
| Nervous system development | 7.31928E-13 |
| Neurotransmitter receptors and postsynaptic signal transmission | 1.26974E-12 |
| regulation of secretion by cell | 5.79412E-12 |
| melanosome | 6.10794E-12 |
| cell body | 1.18428E-11 |
| GTPase activity | 1.82396E-11 |
| Gap junction | 2.66321E-11 |
| synapse organization | 2.85545E-11 |
| dendrite | 3.25009E-11 |
| cellular response to metal ion | 4.48607E-11 |

Note: The categories were obtained from Metascape. * indicates that the category was significant from GCEA using ensemble-based null models. NA indicates that the corresponding or related category cannot be found from GCEA using ensemble-based null models. Abbreviations: GCEA, gene-category enrichment analysis.

# Supplementary Figures


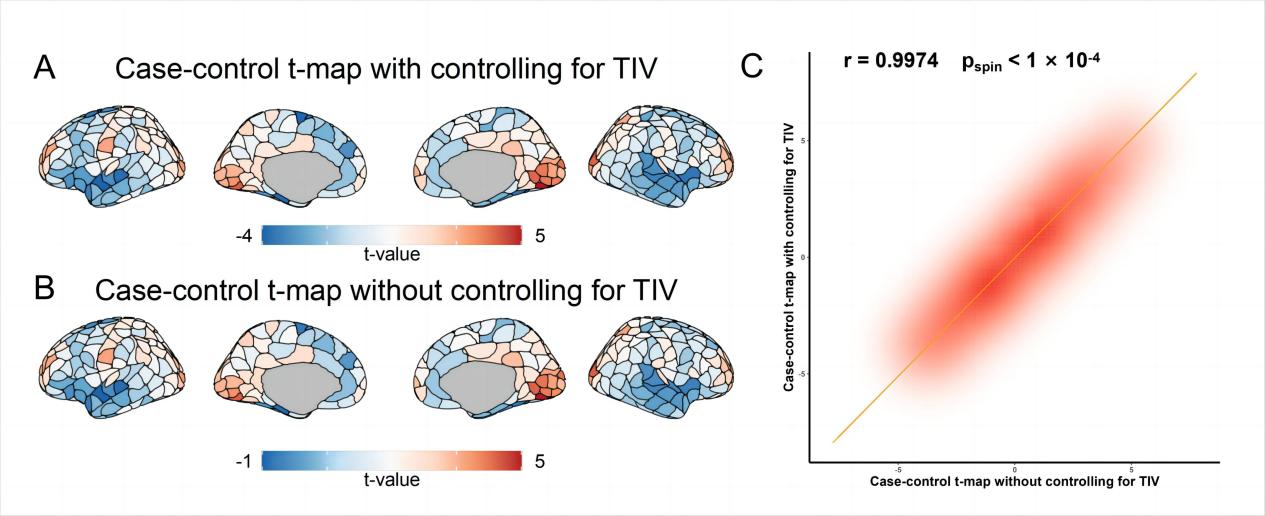


## Figure S1. TIV effect on case-control differences.

(**A**) The case-control t map with controlling for TIV. (**B**) The case-control t map without controlling for TIV (main results). (**C**) The results of spatial correlation analysis between the MSN with and without controlling for TIV. P value was calculated based on spin test. Abbreviations: MSN, morphometric similarity network; TIV, total intracranial volume.


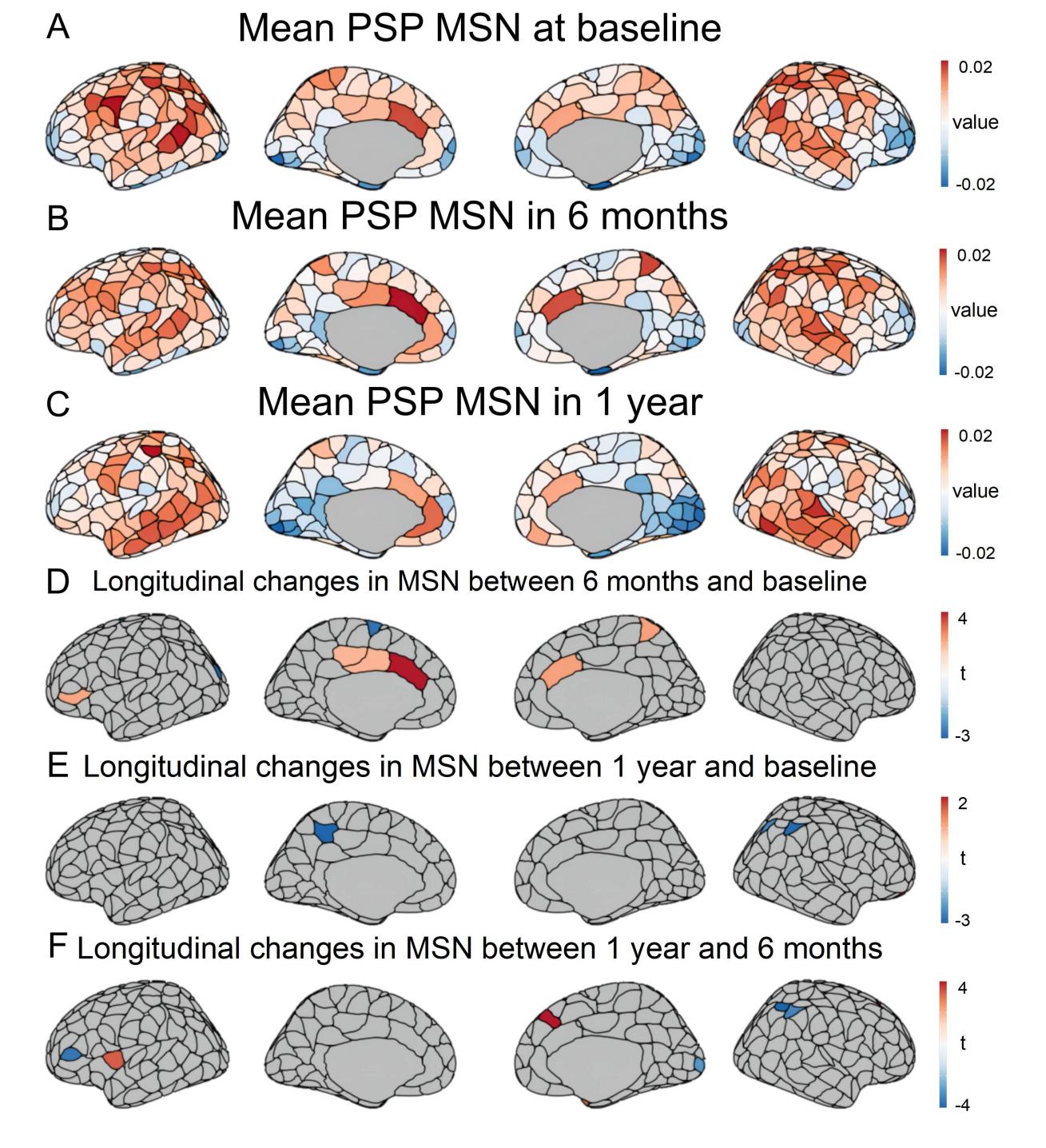


## Figure S2. Longitudinal Study of Morphological Similarity Network Changes.

(**A**-**C**) The mean changes of MSN in PSP patients at three time points: baseline, 6 months, and 1 year. (D-F) Comparison of the longitudinal changes in PSP-related MSN using GLM models, respectively: (**D**) MSN changes in PSP at 6 months and baseline. P < 0.05. (**E**) MSN changes in PSP at 1 year and baseline. P < 0.05. (**F**) MSN changes in PSP at 1 year and 6 months. P < 0.01. All p value without FDR-corrected.


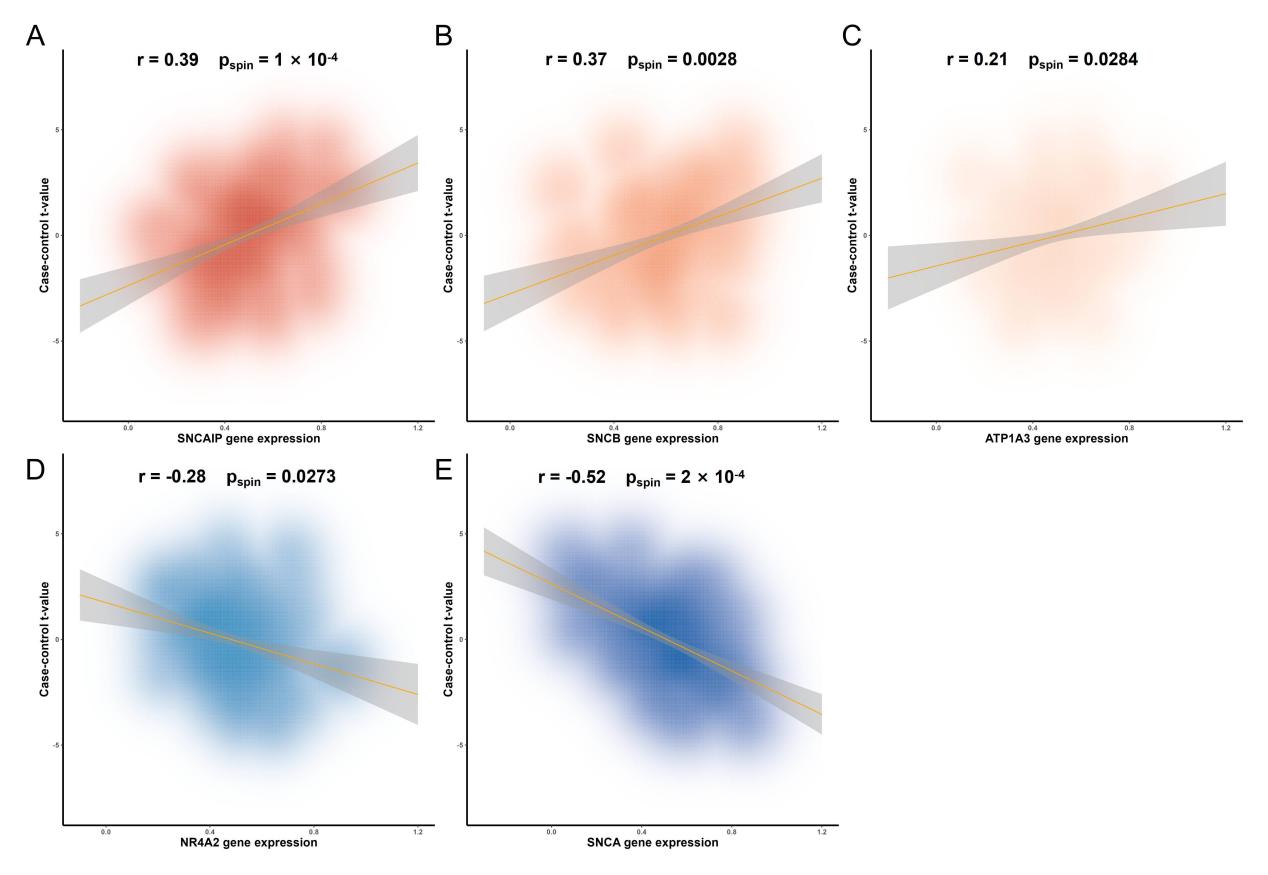


## Figure S3. The significant correlations between the other five PSP-related genes from the AHBA database and the case-control t-map.

Three of five genes were positively correlated with regional changes in the MSN, and two of five genes were negatively correlated with regional changes in the MSN. All p values were derived from spin tests and adjusted by the BH-FDR method. Abbreviations: AHBA, Allen Human Brain Atlas; BH-FDR, Benjamini-Hochberg false discovery rate; MSN, morphometric similarity network; PSP, progressive supranuclear palsy.


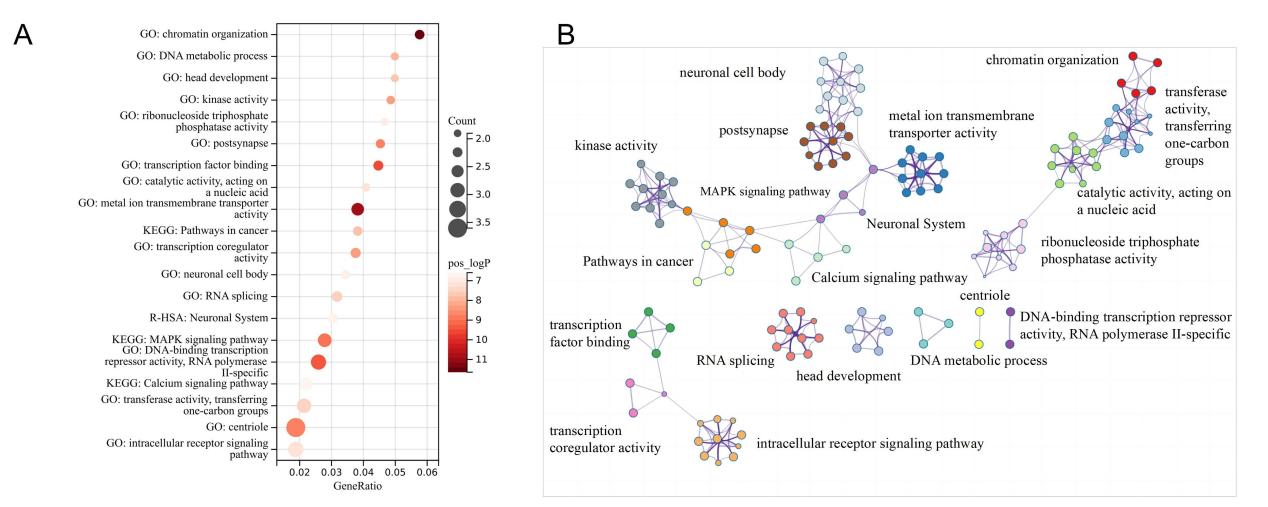


## Figure S4. Enrichment pathways of PLS1+ gene list.

(**A**) Bubble plot of significant enrichment terms. The color of the circle represents -log10 (p-value), and the size of circle represents the number of genes in the same term. (**B**) Visualization of enriched ontology terms. A circle node represents a given term, and its size depends on the number of genes in that term. Circle nodes of the same color belong to the same cluster.


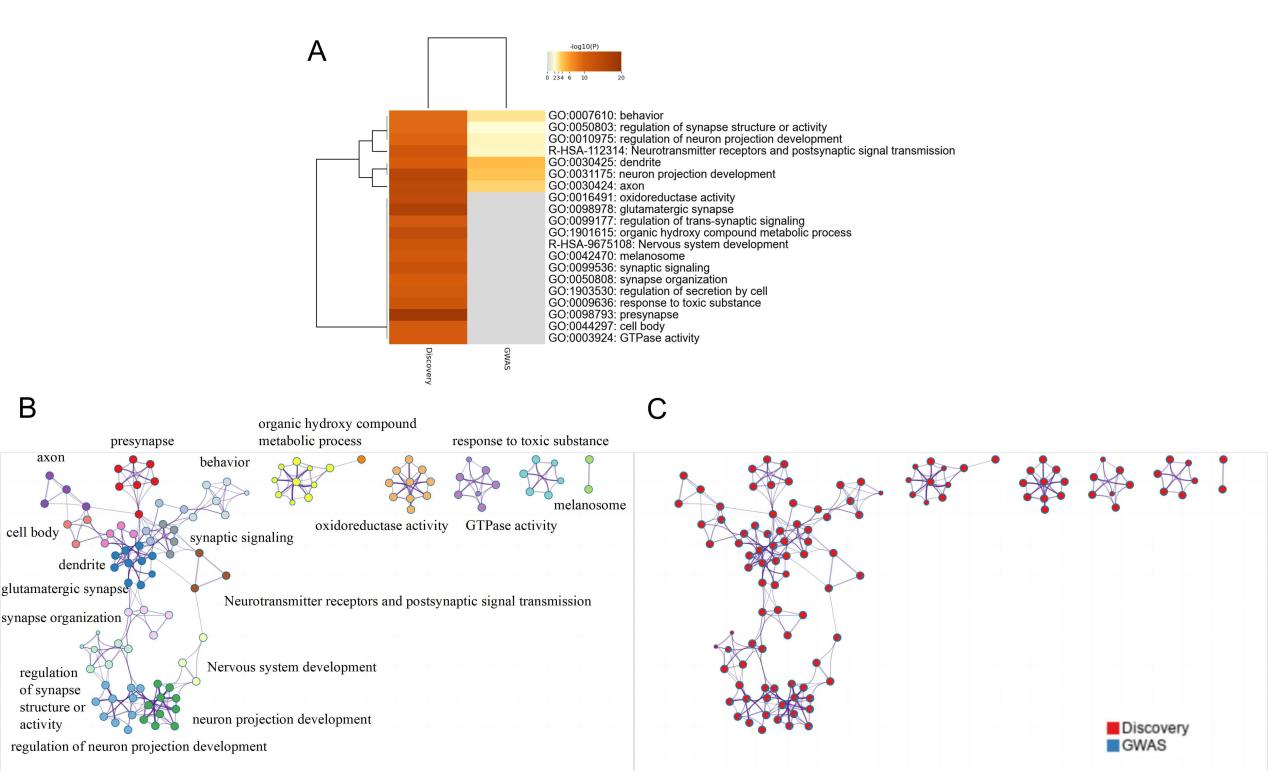


## Figure S5. Shared pathways between the MSN differences-related genes and genes from GWAS.

(**A**) The heatmap cells were colored based on corrected p-values, and the gray cells indicate the lack of enrichment pathways in the corresponding gene list. (**B**) The plot was a subset of ontology terms from A for the PLS1 **−** gene list and the GWAS gene list. (**C**) The shared pathways distribution. The nodes of the shared enrichment network were displayed as pies. Each pie sector was proportional to the number of shared pathways between two different gene lists. Blue was for genes from GWAS, and red was for PLS1 **−** genes. Abbreviations: GO, Gene Ontology; GTPases, nucleotide guanosine tri-phosphatases; GWAS, genome-wide association studies; hsa, the Kyoto encyclopedia of genes and genomes (KEGG)-Homo sapiens; MSN, morphometric similarity network; R-HSA, Reactome-Homo sapiens.


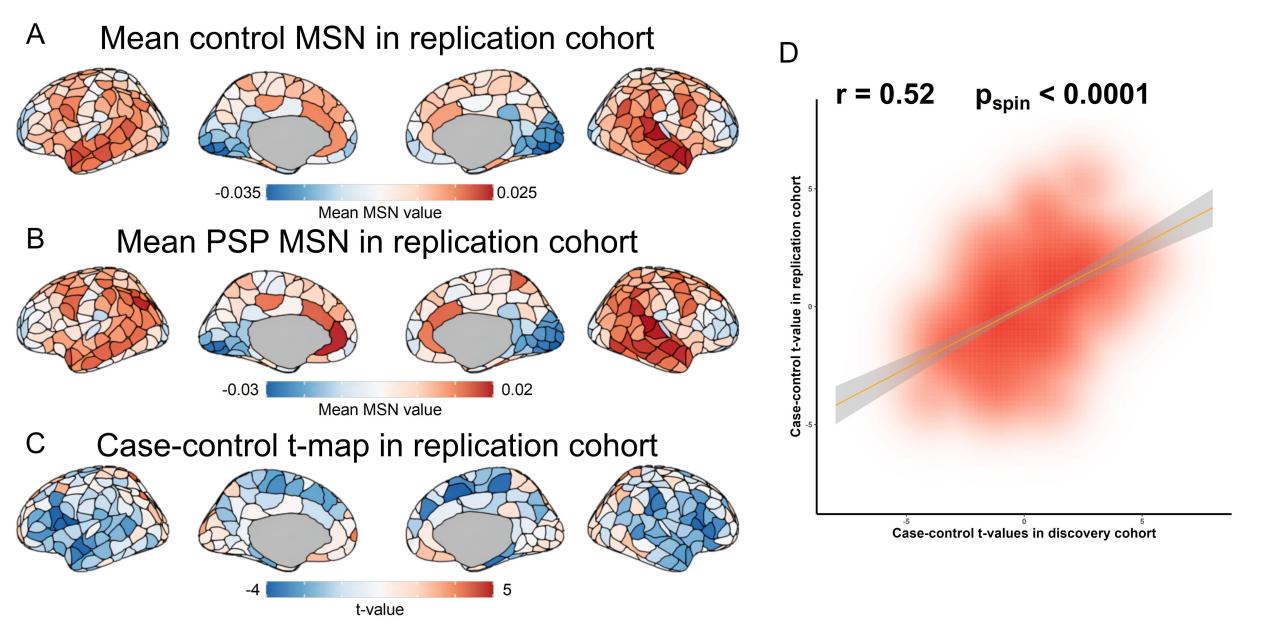


## Figure S6. Replicable regional MSN differences of PSP.

(A) The mean regional MSN of healthy controls in replication cohort. (B) The mean regional MSN of PSP in replication cohort. (C) Regional case-control MSN differences in replication cohort. (D) The results of spatial correlation analysis with the MSN between the discovery cohort and the replication cohort. P value was calculated based on spin test.


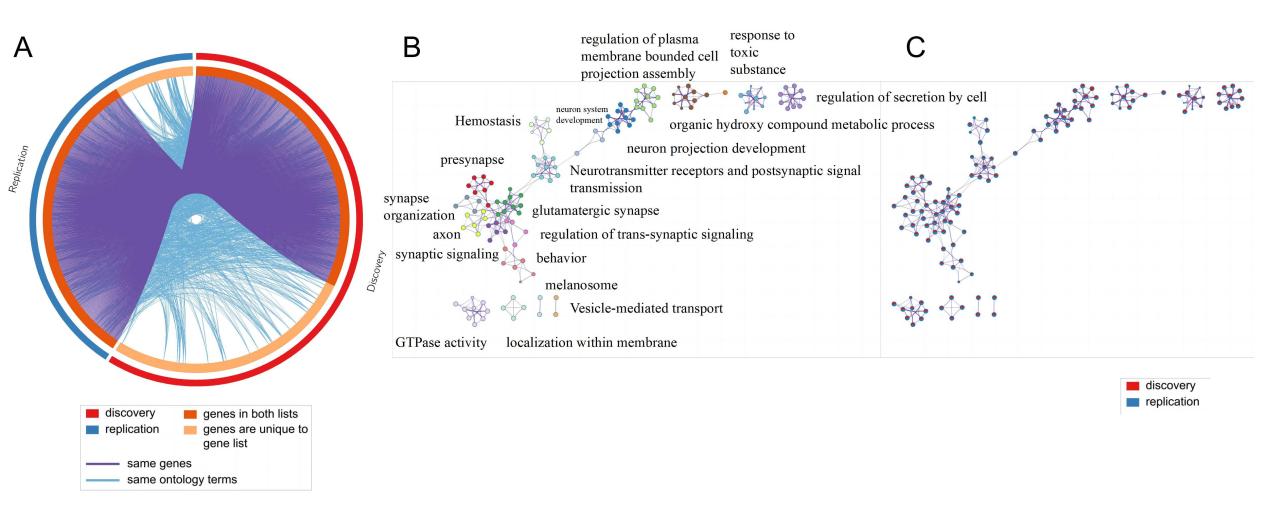


## Figure S7. Reproducibility analysis of the transcriptional enrichment of changes in MSN.

(**A**) gene Circos diagram showing the overlap of the discovery and replication groups. (**B**) A selection of indicative terms from every cluster. (**C**) Identical network for enrichment displayed, featuring its nodes in various pie sections. Every pie sector corresponds to the quantity of identifications derived from a gene list.
